# Supplementary figures and images for: Functional characterization of eicosanoid signaling in Drosophila development
Source: PLoS Genet. 2025 May 9;21(5):e1011705. doi: 10.1371/journal.pgen.1011705 (PMC12088517; doi:10.1371/journal.pgen.1011705)

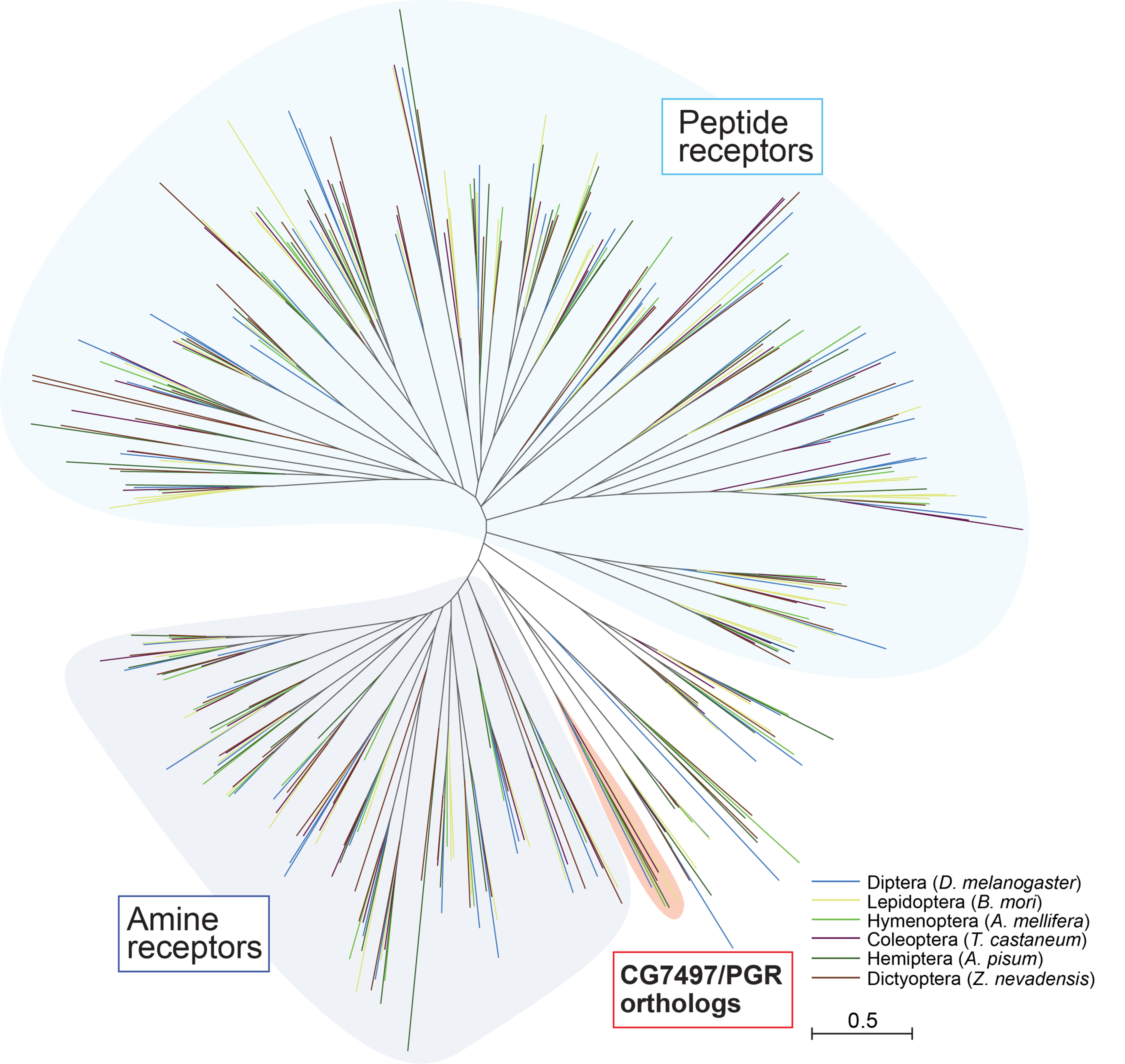

Supplement: S1 Fig — Unrooted maximum-likelihood phylogenetic tree of class A GPCRs in Drosophila melanogaster, Bombyx mori, Apis mellifera, Tribolium castaneum, Acyrthosiphon pisum, and Zootermopsis nevadensis. CG7497/PGR orthologs are conserved in all insect species analyzed. The scale bar indicates an evolutionary distance of 0.5 amino acid substitutions per site. Accession numbers of the receptors analyzed are listed in S2 Table. (TIF) [file pgen.1011705.s001.tif]

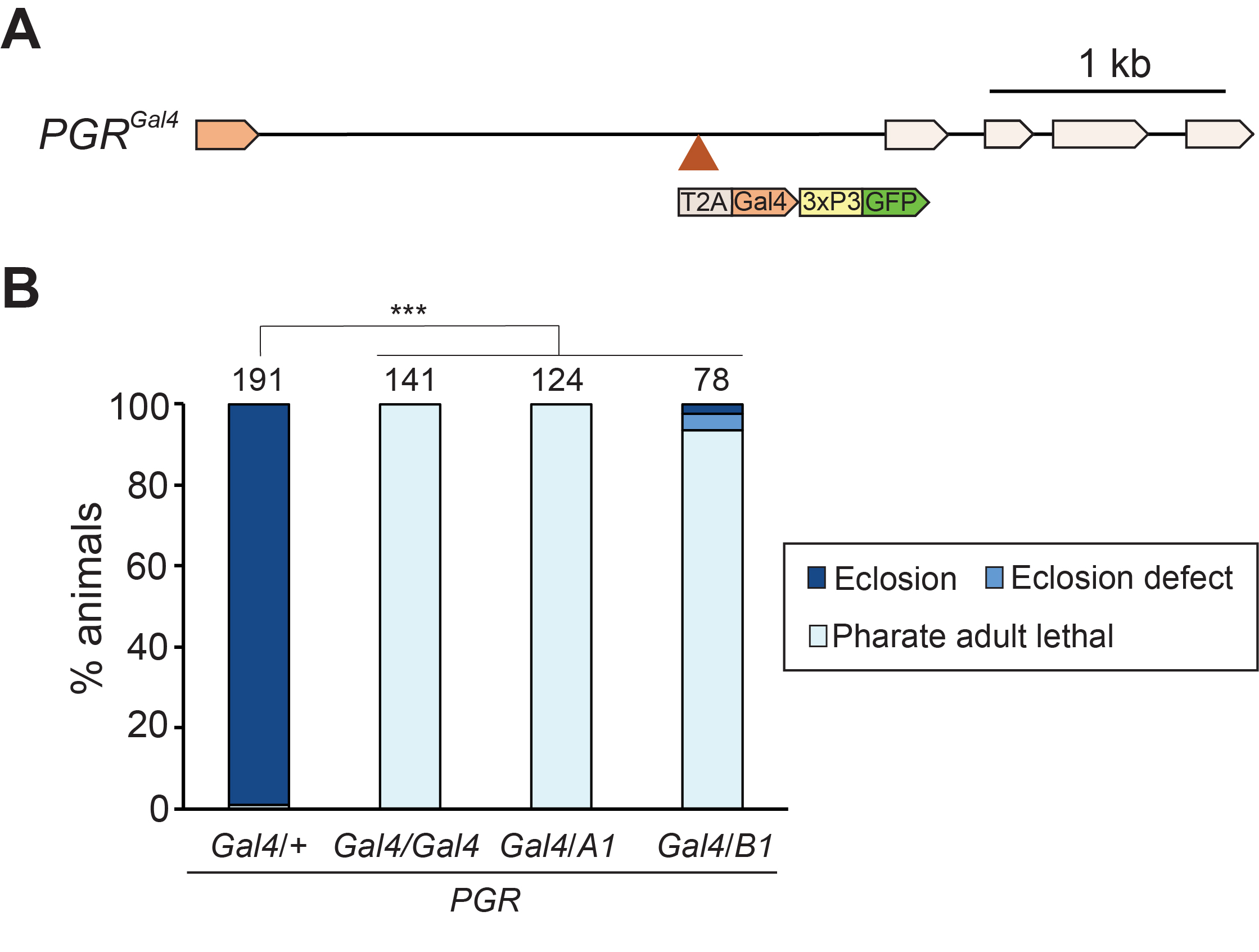

Supplement: S2 Fig — (A) Schematic diagram of the PGRGal4 allele [29]. T2A-Gal4 is inserted in the first intron of PGR along with the 3xP3-GFP marker. (B) Developmental phenotype of the PGRGal4 mutant. Most homozygous and transheterozygous PGR mutants died as pharate adults, indicating the loss of PGR function in PGRGal4 flies. ***p < 0.001 (multiple comparison Chi-square test with Bonferroni correction). Numbers above the bars indicate flies analyzed in each genotype. (TIF) [file pgen.1011705.s002.tif]

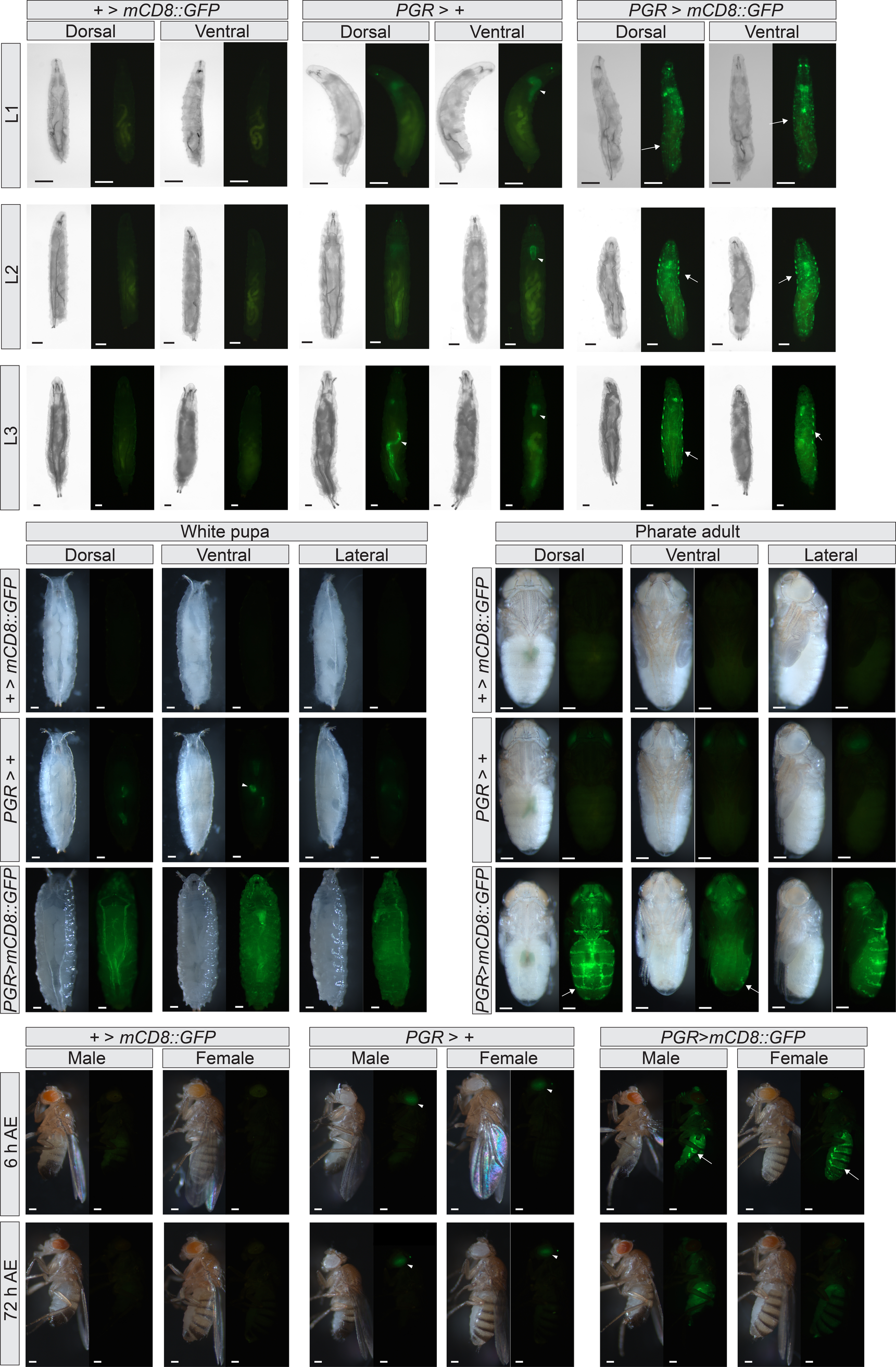

Supplement: S3 Fig — Expression patterns of PGR visualized by PGR-Gal4-driven expression of UAS-mCD8::GFP. Strong GFP signals were observed in the tracheae in all developmental stages tested. They were also observed in oenocytes in larvae, pharate adults, and newly emerged adults as indicated by arrows. Due to the 3xP3-GFP marker in the PGR-Gal4 line, background GFP signals are observed in the larval CNS, larval gut, and adult eyes as indicated by arrowheads. Detailed tissue-specific expression patterns are shown in Fig 2A. L1–L3, 1st, 2nd, and 3rd instar larvae; AE, after eclosion. Scale bars: 200 µm. (TIF) [file pgen.1011705.s003.tif]

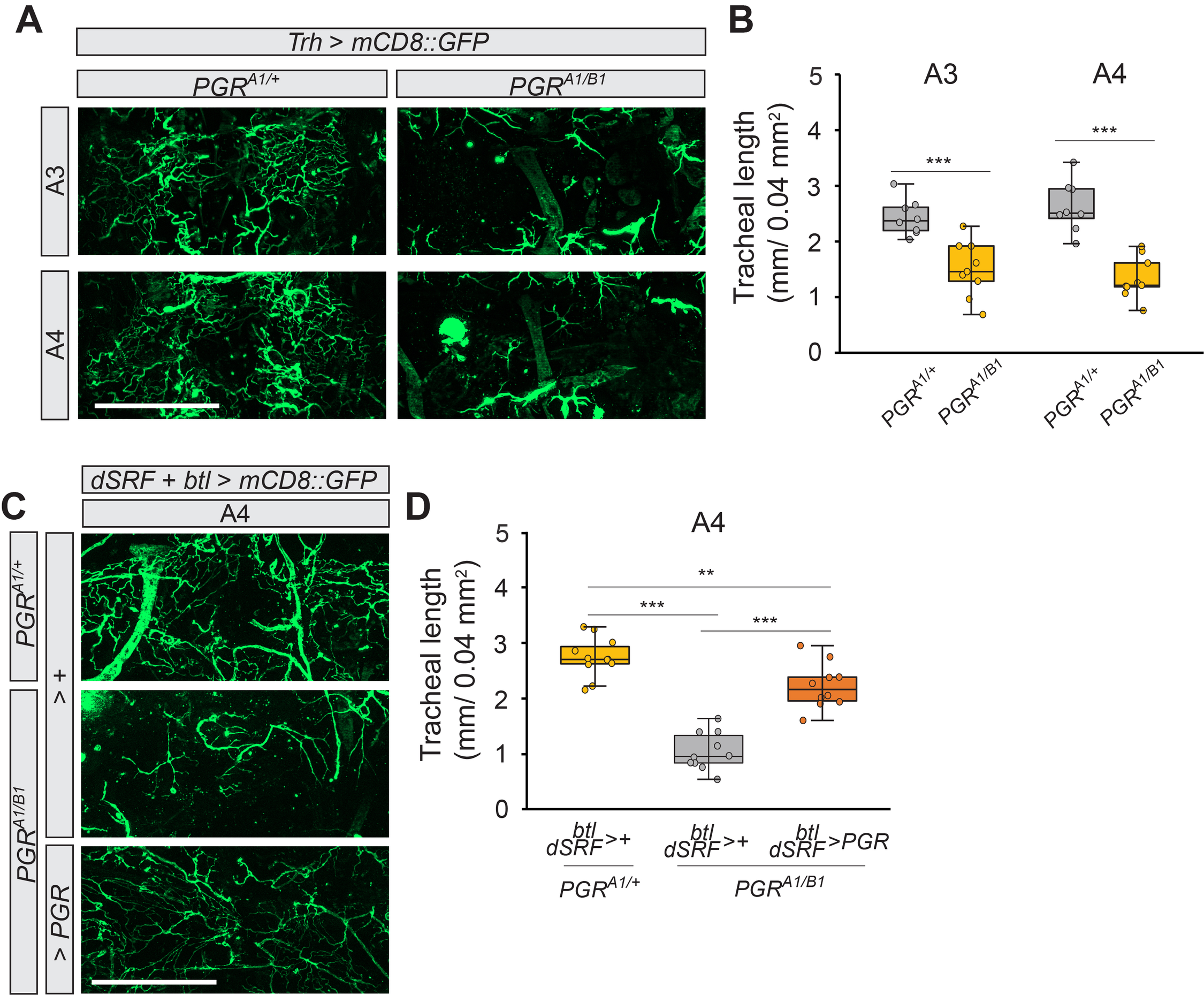

Supplement: S4 Fig — (A) Pupal abdominal tracheae in heterozygous and transheterozygous PGR mutants visualized by Trh-Gal4-driven UAS-mCD8::GFP expression at 72 hours after puparium formation (APF). (B) Total tracheal length in the third (A3) and fourth (A4) abdominal segments in the selected area as visualized by Trh-Gal4-driven UAS-mCD8::GFP expression. Transheterozygous mutants showed defective tracheal development in both segments. n = 8–9. ***p < 0.01 (Student’s t-test). (C) Pupal abdominal tracheae in heterozygous and transheterozygous PGR mutants visualized by btl-Gal4- and dSRF-Gal4-driven UAS-mCD8::GFP expression at 72 hours APF. Overexpression of PGR in the tracheae and TTCs partially rescued underdeveloped tracheoles in PGR mutants. (D) Total tracheal length in A4 in the selected area as visualized by btl-Gal4- and dSRF-Gal4-driven UAS-mCD8::GFP expression. Tracheal length in A3 was not measured due to excessive GFP signals in A3 driven by dSRF-Gal4. n = 10–11. **p < 0.1, ***p < 0.01 (Tukey’s honestly significant difference test). Scale bars: 200 µm. (TIF) [file pgen.1011705.s004.tif]

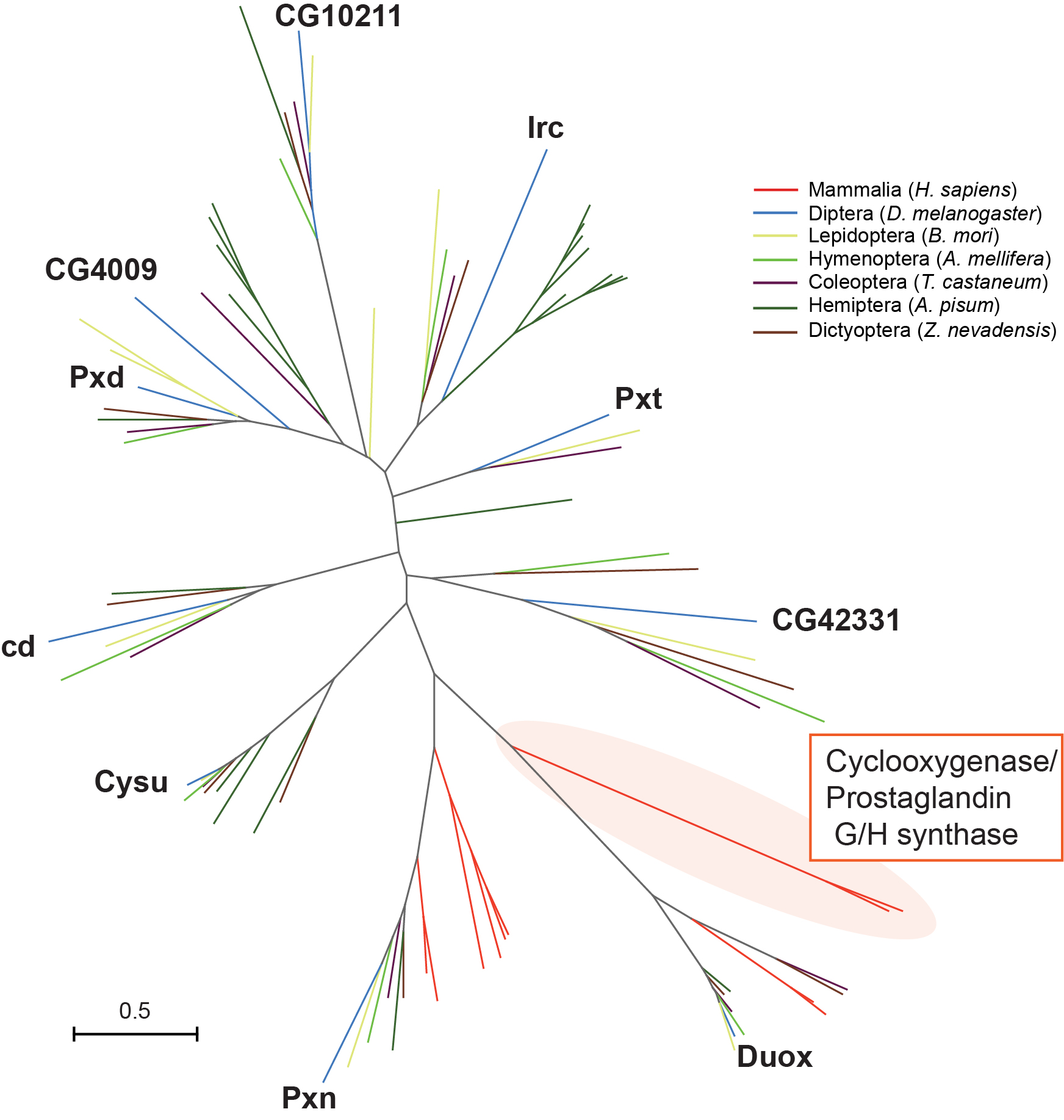

Supplement: S5 Fig — Unrooted maximum-likelihood phylogenetic tree of heme peroxidases in Homo sapiens, Drosophila melanogaster, Bombyx mori, Apis mellifera, Tribolium castaneum, Acyrthosiphon pisum, and Zootermopsis nevadensis. Branches are color-coded for different species. Cyclooxygenases (PGG/H synthases) in H. sapiens are highlighted. The scale bar indicates an evolutionary distance of 0.5 amino acid substitutions per site. Accession numbers of the enzymes analyzed are listed in S3 Table. (TIF) [file pgen.1011705.s005.tif]

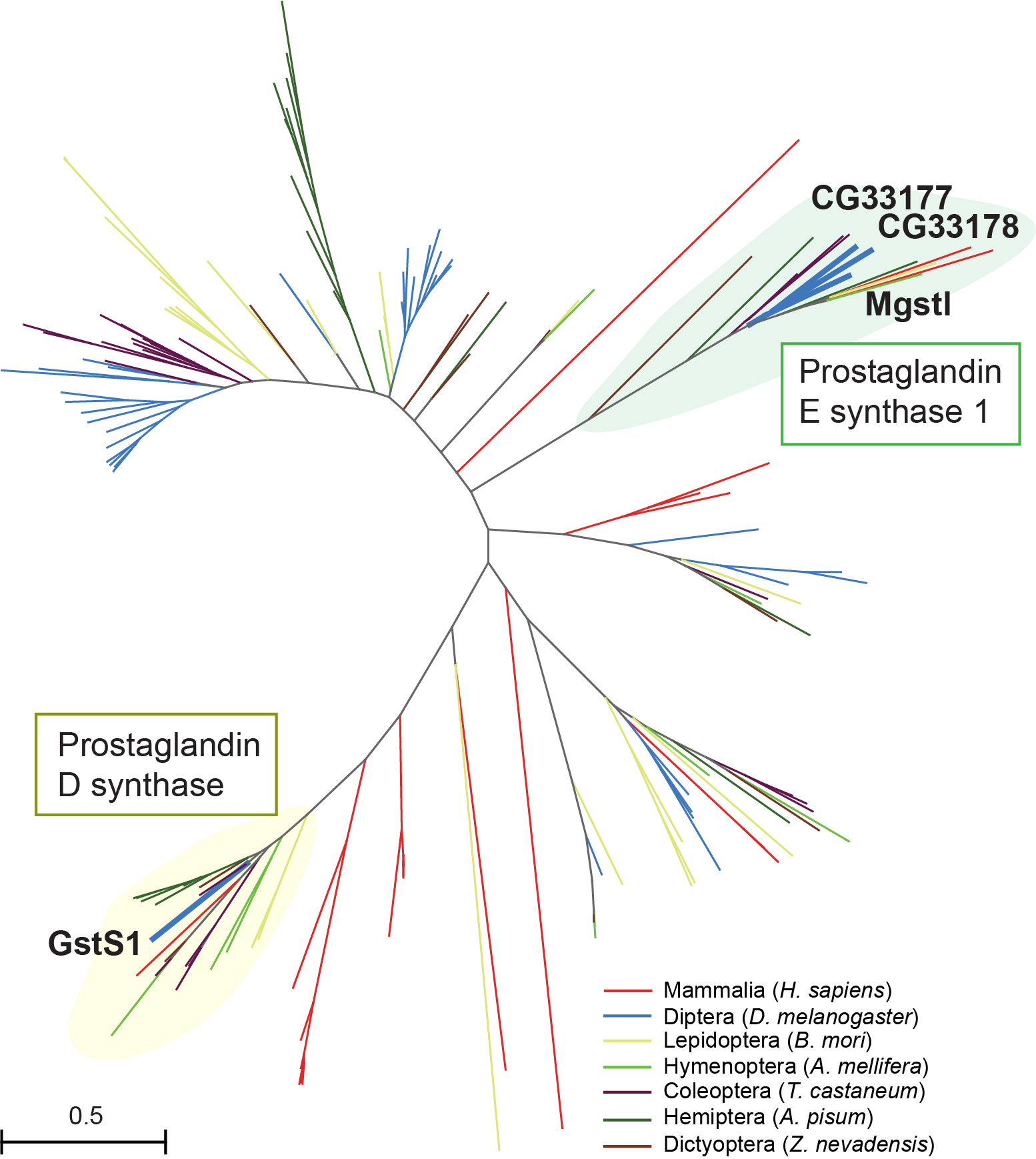

Supplement: S6 Fig — Unrooted maximum-likelihood phylogenetic tree of glutathione S-transferases in Homo sapiens, Drosophila melanogaster, Bombyx mori, Apis mellifera, Tribolium castaneum, Acyrthosiphon pisum, and Zootermopsis nevadensis. Branches are color-coded for different species. Clades that include PGD synthase and PGE synthase 1 in H. sapiens are highlighted. The scale bar indicates an evolutionary distance of 0.5 amino acid substitutions per site. Accession numbers of the enzymes analyzed are listed in S4 Table. (TIF) [file pgen.1011705.s006.tif]

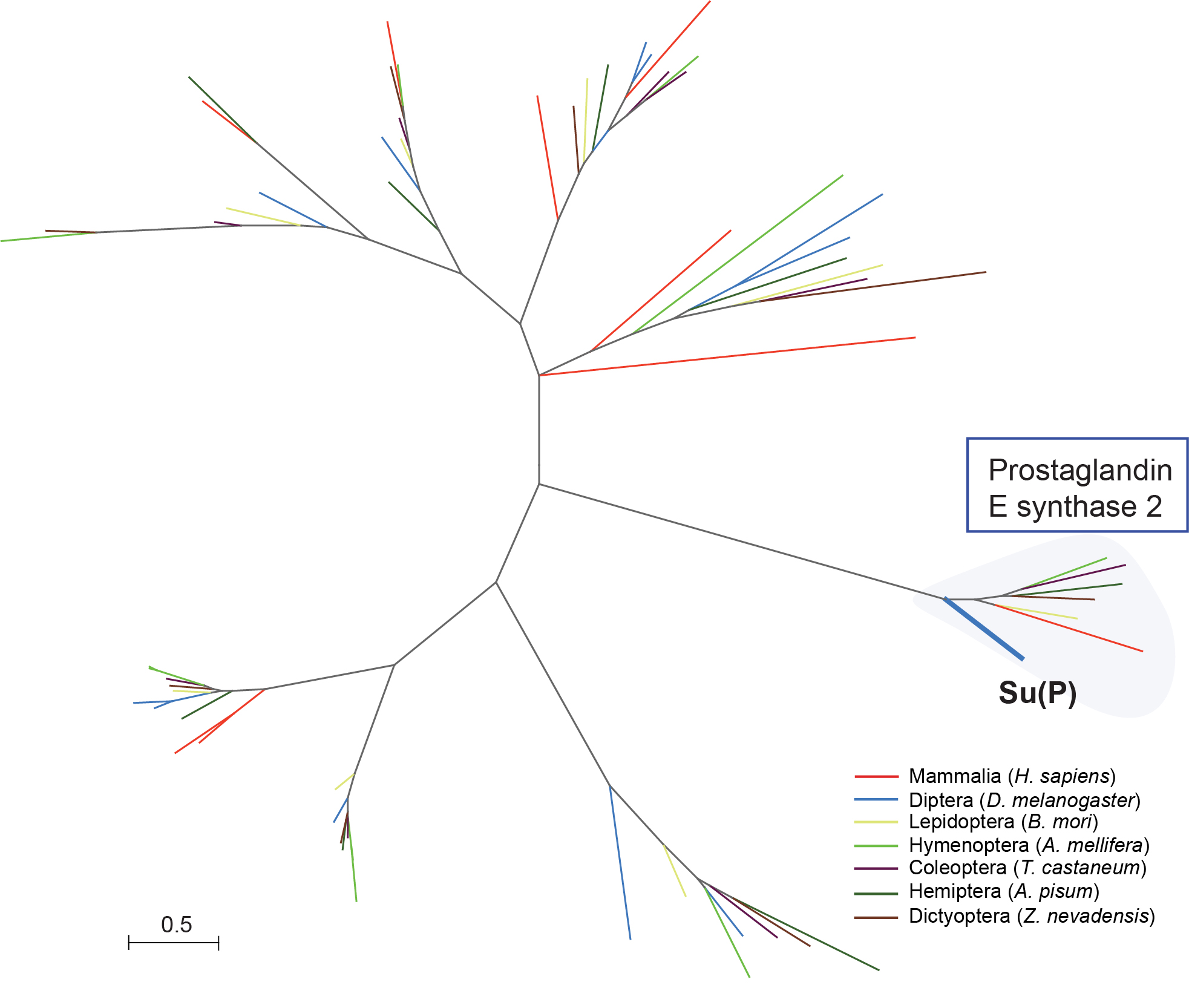

Supplement: S7 Fig — Unrooted maximum-likelihood phylogenetic tree of glutaredoxin domain-containing proteins in Homo sapiens, Drosophila melanogaster, Bombyx mori, Apis mellifera, Tribolium castaneum, Acyrthosiphon pisum, and Zootermopsis nevadensis. Branches are color-coded for different species. The clade that includes PGE synthase 2 in H. sapiens is highlighted. The scale bar indicates an evolutionary distance of 0.5 amino acid substitutions per site. Accession numbers of the enzymes analyzed are listed in S5 Table. (TIF) [file pgen.1011705.s007.tif]

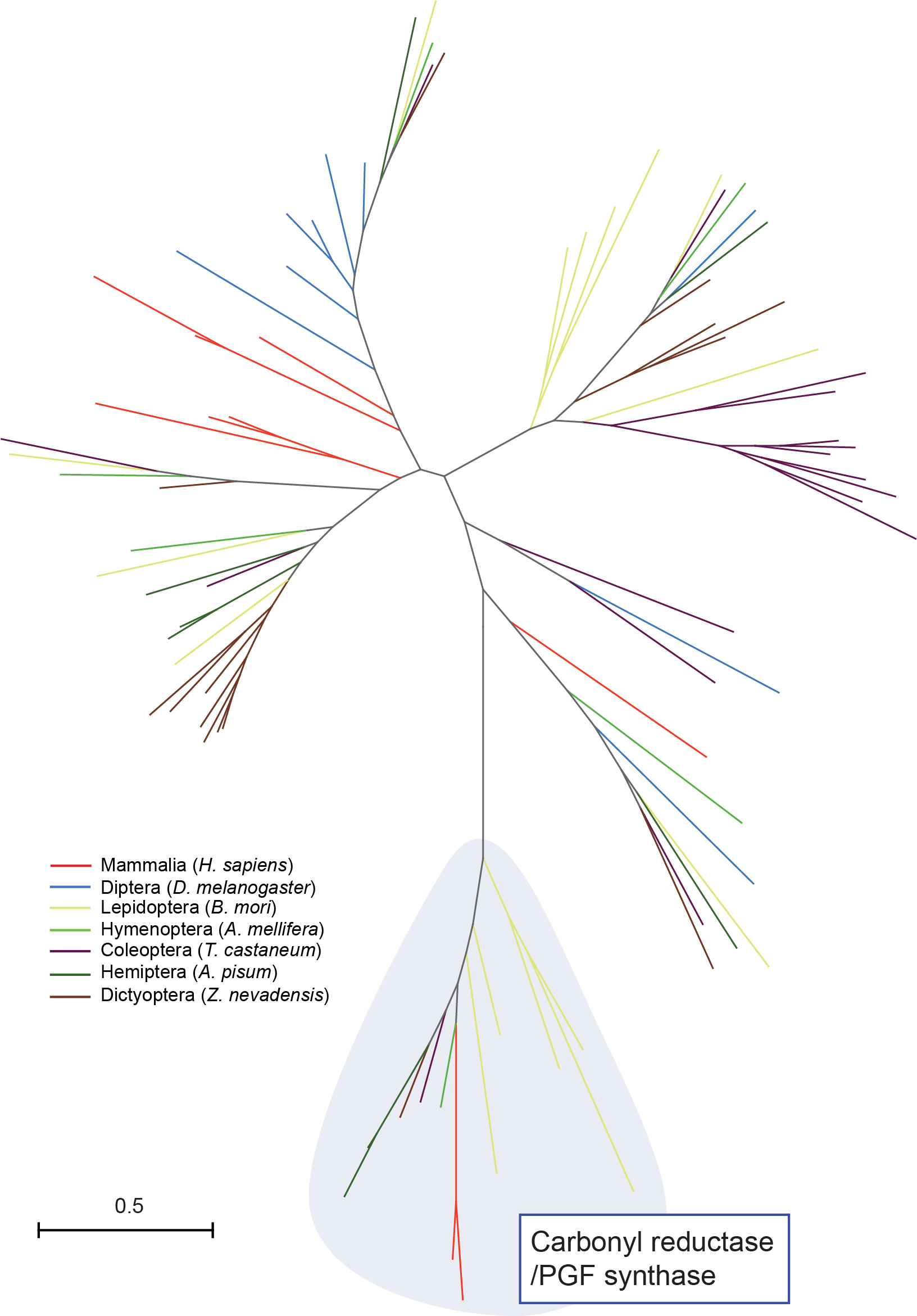

Supplement: S8 Fig — Unrooted maximum-likelihood phylogenetic tree of carbonyl reductases in Homo sapiens, Drosophila melanogaster, Bombyx mori, Apis mellifera, Tribolium castaneum, Acyrthosiphon pisum, and Zootermopsis nevadensis. Branches are color-coded for different species. The clade that includes carbonyl reductase 1 (PGF synthase) in H. sapiens is highlighted. The scale bar indicates an evolutionary distance of 0.5 amino acid substitutions per site. Accession numbers of the enzymes analyzed are listed in S6 Table. (TIF) [file pgen.1011705.s008.tif]

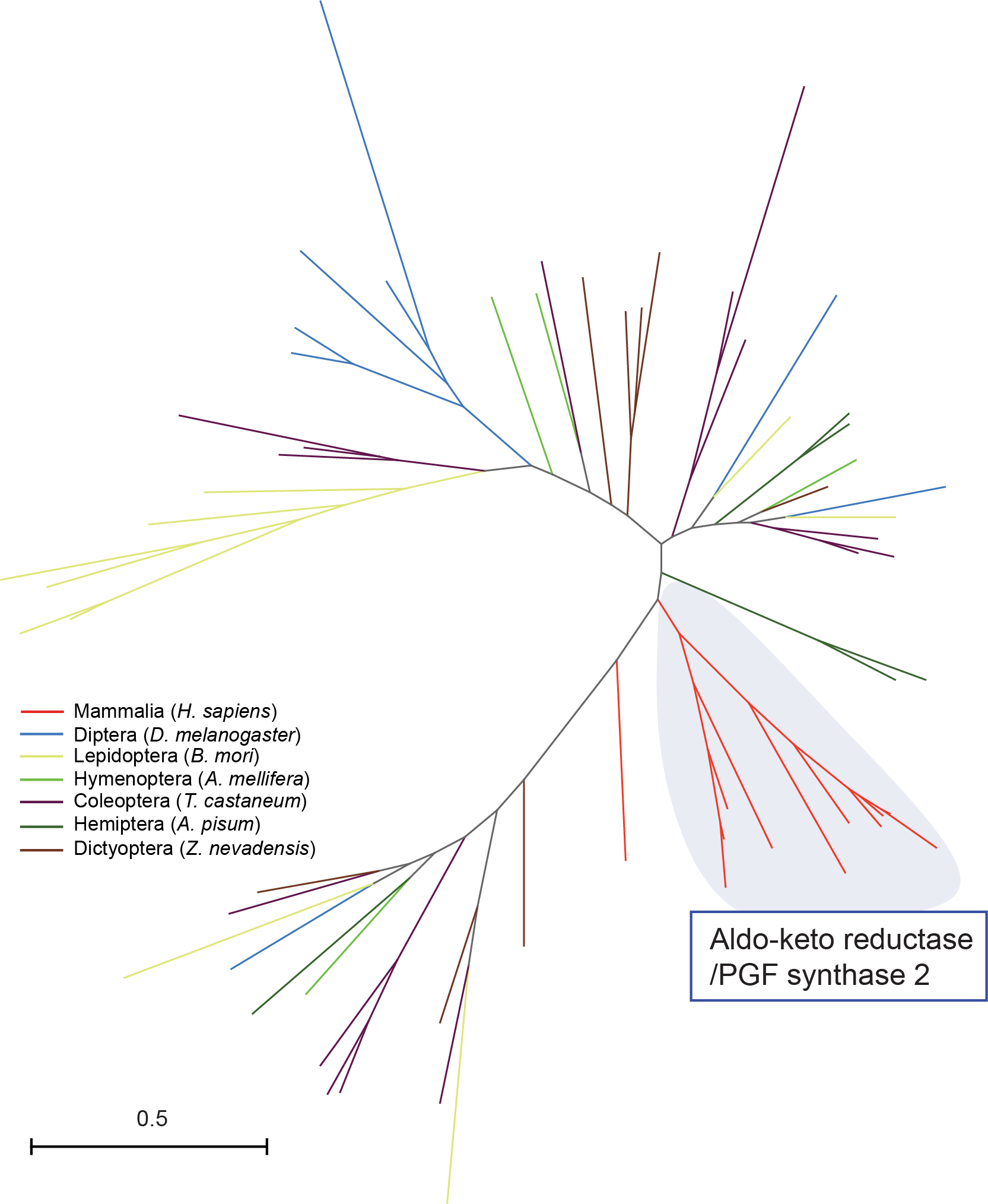

Supplement: S9 Fig — Unrooted maximum-likelihood phylogenetic tree of aldo-keto reductases in Homo sapiens, Drosophila melanogaster, Bombyx mori, Apis mellifera, Tribolium castaneum, Acyrthosiphon pisum, and Zootermopsis nevadensis. Branches are color-coded for different species. The clade that includes aldo-keto reductase (PGF synthase 2) in H. sapiens is highlighted. The scale bar indicates an evolutionary distance of 0.5 amino acid substitutions per site. Accession numbers of the enzymes analyzed are listed in S7 Table. (TIF) [file pgen.1011705.s009.tif]

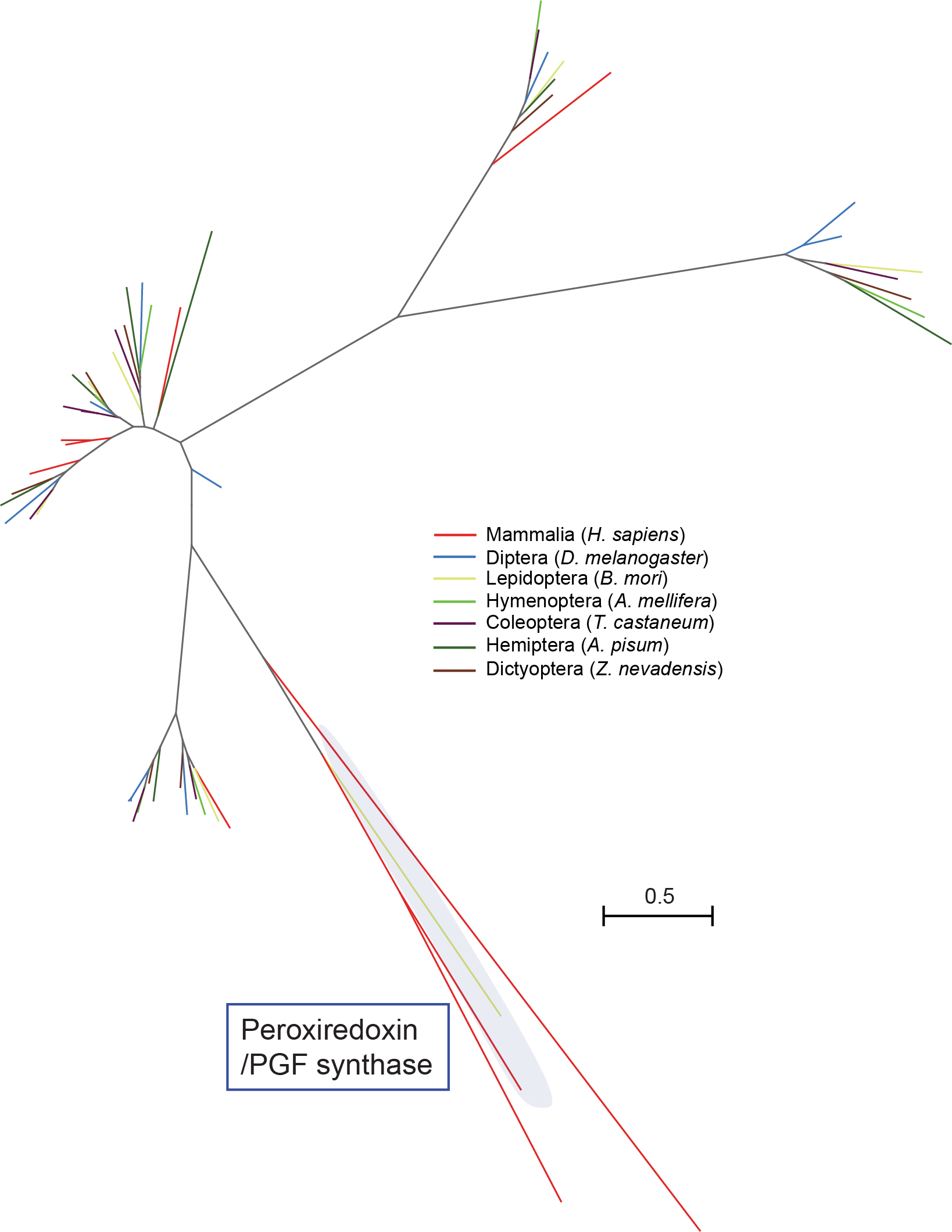

Supplement: S10 Fig — Unrooted maximum-likelihood phylogenetic tree of peroxiredoxins in Homo sapiens, Drosophila melanogaster, Bombyx mori, Apis mellifera, Tribolium castaneum, Acyrthosiphon pisum, and Zootermopsis nevadensis. Branches are color-coded for different species. The clade that includes peroxiredoxin (PGF synthase) in H. sapiens is highlighted. The scale bar indicates an evolutionary distance of 0.5 amino acid substitutions per site. Accession numbers of the enzymes analyzed are listed in S8 Table. (TIF) [file pgen.1011705.s010.tif]

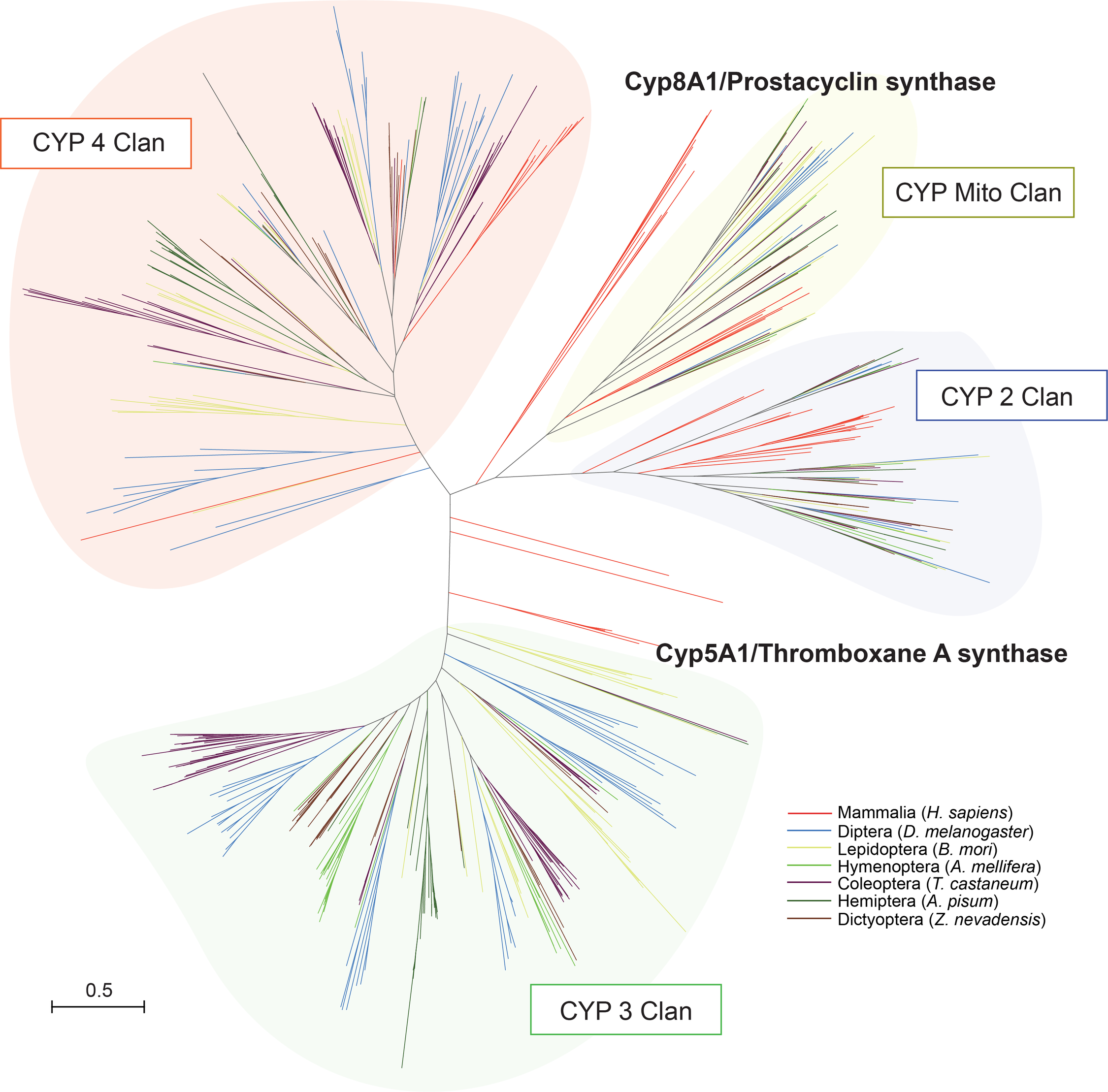

Supplement: S11 Fig — Unrooted maximum-likelihood phylogenetic tree of cytochrome P450 enzymes in Homo sapiens, Drosophila melanogaster, Bombyx mori, Apis mellifera, Tribolium castaneum, Acyrthosiphon pisum, and Zootermopsis nevadensis. Branches are color-coded for different species. The four major CYP clans in insects are highlighted. There are no orthologous enzymes of Cyp5A1 or Cyp8A1 in insects. The scale bar indicates an evolutionary distance of 0.5 amino acid substitutions per site. Accession numbers of the enzymes analyzed are listed in S9 Table. (TIF) [file pgen.1011705.s011.tif]

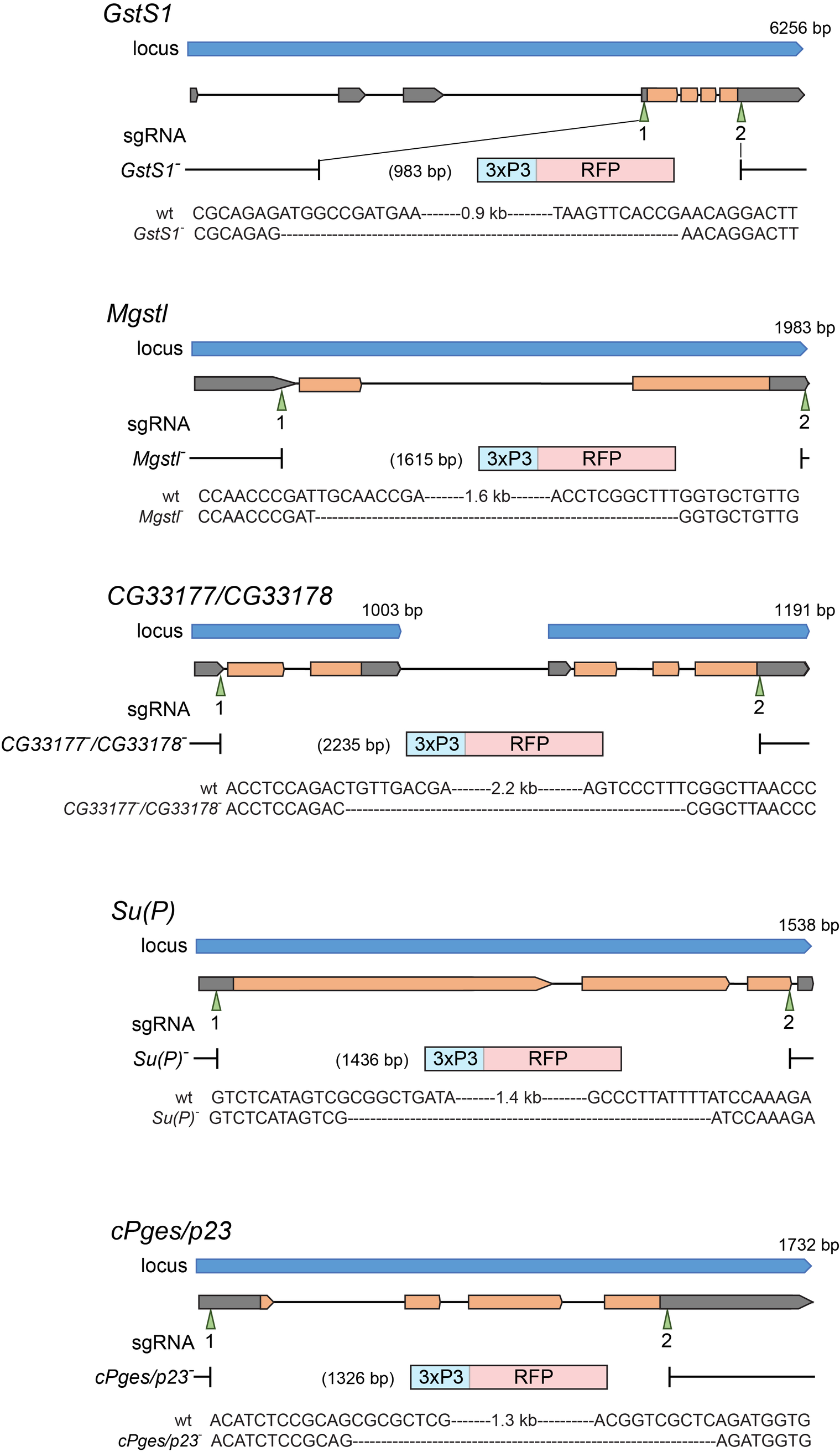

Supplement: S12 Fig — Mutagenesis was conducted by CRISPR-Cas9-based homologous recombination to insert the 3xP3-RFP sequence into each target site. Two single guide RNAs (sgRNAs) were designed for each target to delete entire coding sequences shown in orange. Genomic sequences of the mutants are provided in S2–S6 Documents. (TIF) [file pgen.1011705.s012.tif]

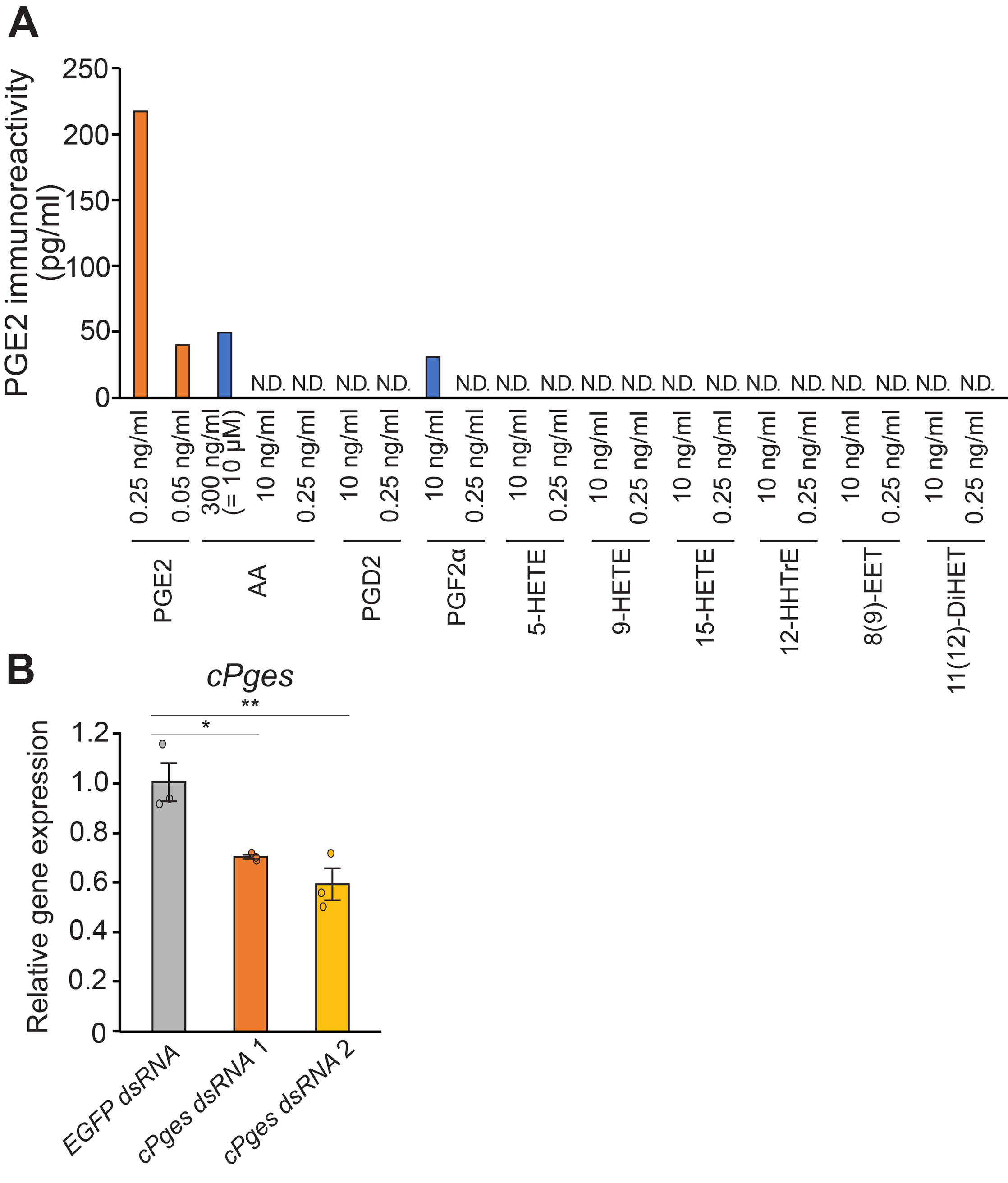

Supplement: S13 Fig — (A) Cross-reactivity of the anti-PGE2 antibody used in the PGE2 ELISA system. Serial dilutions of AA and various eicosanoids previously detected in the Drosophila hemolymph after AA injection [22] were tested for their potential cross-reactivity. AA and PGF2α have 0.017% and 0.25% cross-reactivity, respectively. HETE, hydroxyeicosatetraenoic acid; HHTrE, hydroxyheptadecatrienoic acid; EET, eicosatrienoic acid; DiHET, dihydroxyeicosatetraenoic acid. (B) Relative expression levels of cPges/p23 in S2 cells treated with dsRNA for 3 days. cPges/p23 mRNA levels were downregulated in cPges/p23 RNAi cells as compared to the negative control (EGFP RNAi). Expression levels are normalized by the levels of a reference gene, rp49, in the same cDNA samples. n = 3. *p < 0.05, ** < 0.01 (Dunnett’s test vs EGFP RNAi). (TIF) [file pgen.1011705.s013.tif]

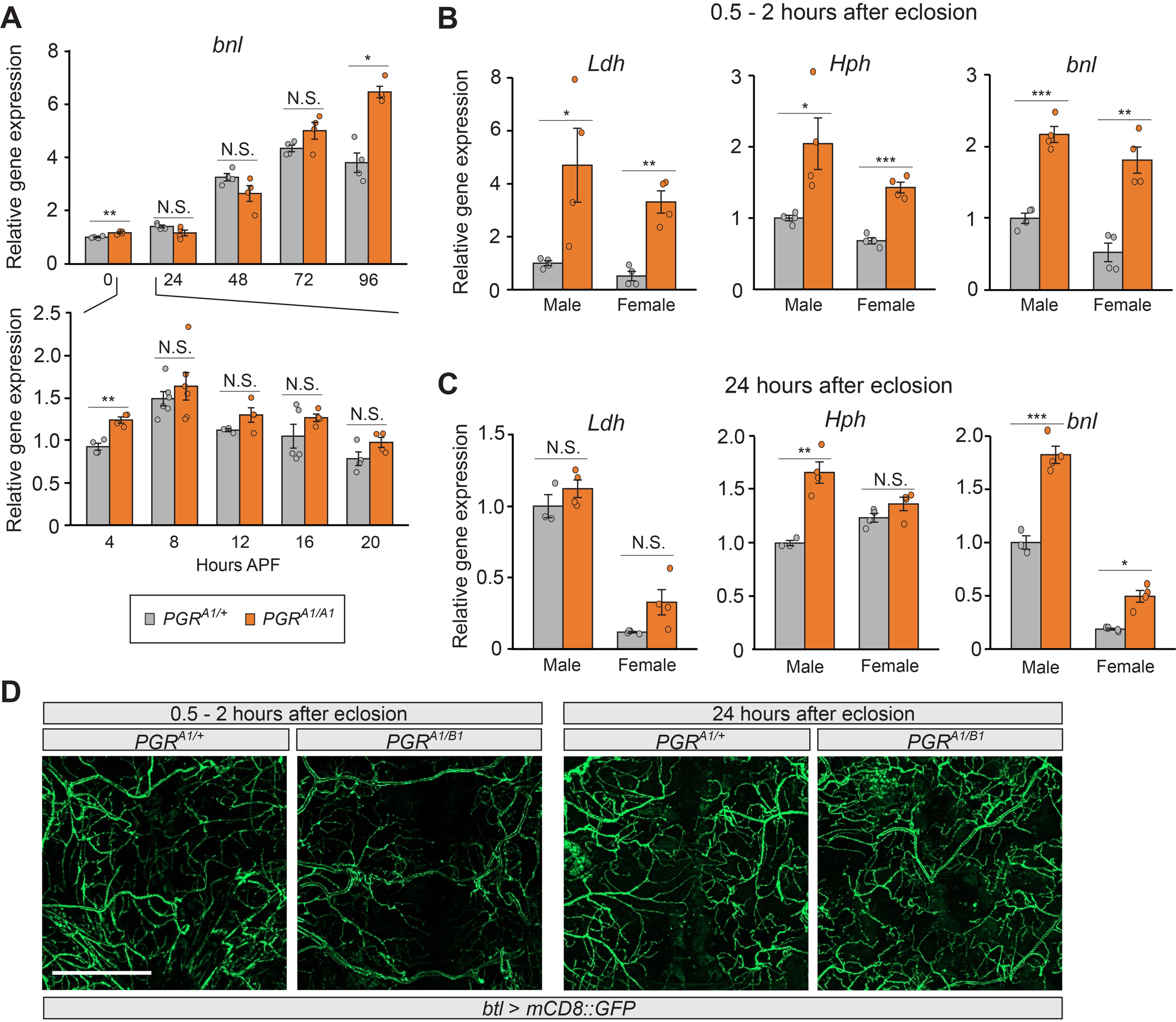

Supplement: S14 Fig — (A) Relative expression levels of branchless (bnl) in PGR mutants from 0 to 96 hours after puparium formation (APF). Insects pupated about 12 hours APF. Homozygous mutant pupae did not show significantly higher expression of bnl until 96 hours APF. (B, C) Relative expression levels of hypoxia response genes (Ldh and Hph) and bnl in adult PGR mutants rescued by high oxygen supply during pupa-adult development. Eclosed flies were transferred to the normal oxygen condition within 2 hours after eclosion and kept there for 30 min (B) or 24 hours (C) before RNA extraction. Rescued PGR mutant flies express high levels of Ldh and Hph immediately after eclosion, which decreases within 24 hours. In contrast, bnl continues to be highly expressed in PGR mutant flies 24 hours after eclosion. Expression levels are normalized by the levels of a reference gene, rp49, in the same cDNA samples and shown as relative to PGRA1/+ at 0 hours APF. n = 3–4. *p < 0.05, **p < 0.01, ***p < 0.001 (Student’s t-test vs PGRA1/+). (D) Adult tracheal development in PGR heterozygous and transheterozygous mutants raised under 40% oxygen during pupa-adult development. Abdominal tracheae in adult males were visualized by btl-Gal4-driven UAS-mCD8::GFP expression within 2 hours or 24 hours after eclosion. In newly emerged PGR transheterozygous mutants, the adult tracheal system was underdeveloped, which recovered to the level of the control heterozygous mutants within 24 hours after eclosion. Scale bar: 200 µm. (TIF) [file pgen.1011705.s014.tif]

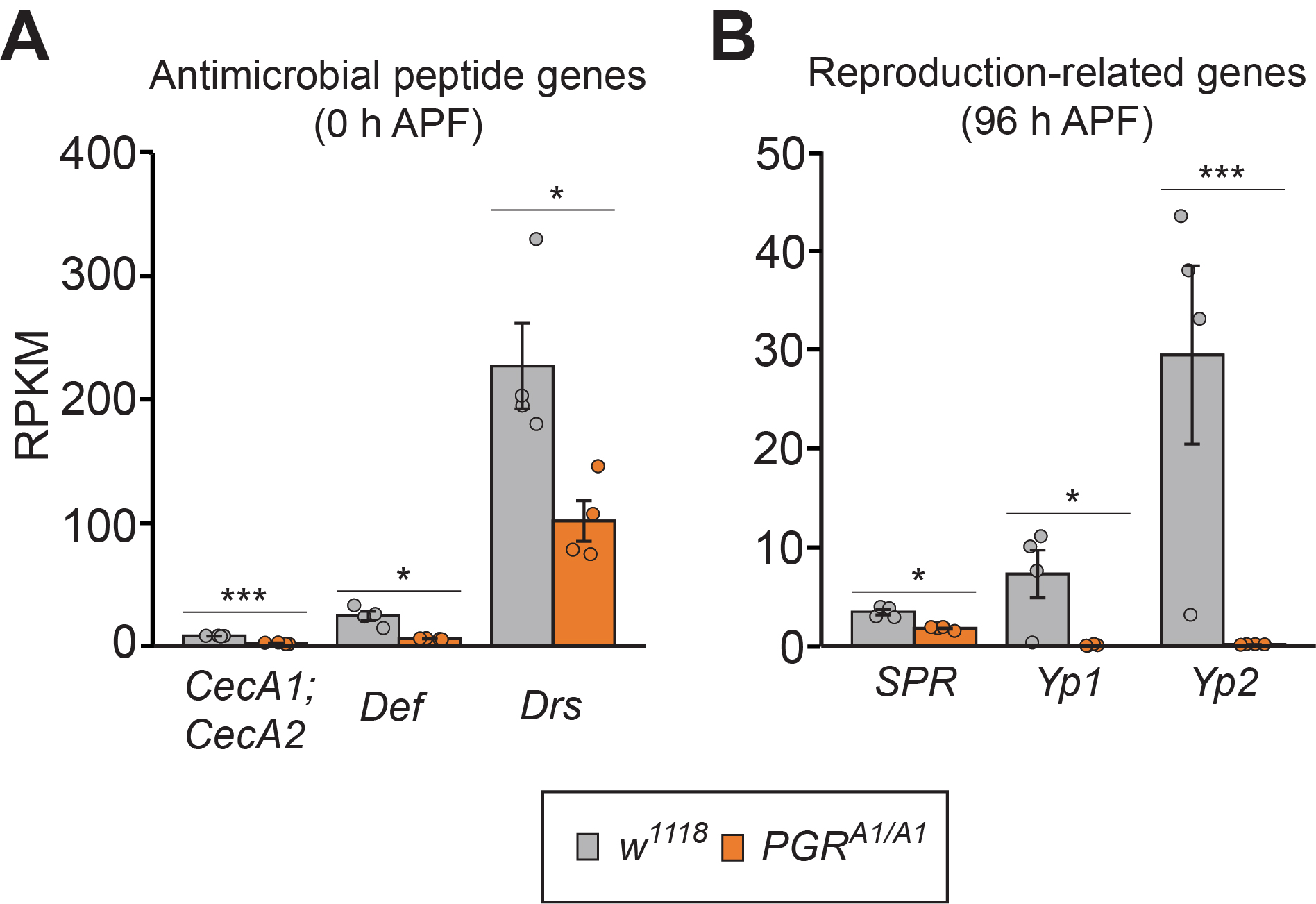

Supplement: S15 Fig — Expression of antimicrobial peptide genes (A) and reproduction-related genes (B) in w1118 control and the PGR mutant based on RNA-seq data. Expression levels of Cecropin A1 and A2 (CecA1 and CecA2), Defensin (Def), and Drosomycin (Drs) in the PGR mutant were significantly lower than control at 0 hours after puparium formation (APF). Expression of Sex peptide receptor (SPR), Yolk protein 1 (Yp1), and Yp2 in the PGR mutant was significantly downregulated at 96 hours APF. Expression levels are shown as reads per kilobase of transcript per million mapped reads (RPKM). *p < 0.05, ***p < 0.001 (Student’s t-test). (TIF) [file pgen.1011705.s015.tif]
