# Supplementary material for: Functional characterization of eicosanoid signaling in Drosophila development
Source: PLoS Genet. 2025 May 9;21(5):e1011705. doi: 10.1371/journal.pgen.1011705 (PMC12088517; doi:10.1371/journal.pgen.1011705)
Supplement: S1 Document — (DOCX) [file pgen.1011705.s029.docx]

Intron : xxxx

Exon : xxxx

CDS : xxxx

gRNA : xxxx

>CG7497/PGR

GCGTGGATGTGGGTGGCCGCTTCGCTTGGGGGTGTCTATGTGCTGTATGCTACATCCGATGCGATCCGACCGGCGGCGGCGCCAGCCGAACTGGCCTCGCTCCGAACTGGCCCCTACCACCACCCCCACGTGTATATGATGGCTTGGATAGCTTGGAGCTATGGGCAGCAGCCGACGAGTGCTCTGCTCGGCACGGTCGGCAGTCATTCTAAATCGACGCCTGGCTGACTGCGGACGCGCTCCTCGGATCGAATCGAATCGCCTTCGAATTGGTCGGTCGTTGGTTGATCAAGTGTCGCGTGCGCTAATCATTAATTAAGTGTCTTAGGAAAAAAGTCCCAATTTGGCTATCGAAACGGGTTTCCATCTACCAGTGCATTTGCGAGCTGCCTTGCCTTTGCGGCAGGCTCATTTATGAAAAAGAAATATCGTTTGCGGCCAGTTAGATTTTCACCTGAATCACCTGCAATCGAACGCAATTATCATACCGGCAAAATGGAAACCACAACACCTGTGCTCGACCTGTTGATGCCGCACAACTCAACTACTGTGGCGCCTCCGAAAGCGCTCTATGCAAATCGAAATCGCCTGATTATTGGTGTTATTATCATGGTTCTGGGCGTTTTTGGCAATTCGCTGGCCCTTTTCATTTTGGCGCGCAAGAAGCTCAACAAAAACAGCAAATACACGCTCATGCTGCGGTAAGTGCAGCTATGCATAGTGGAAATAATAATATATTAATGCAAAAAGTAGGTTTTGCTTGCCCTAAAATCTTTCAAAATGTACCCATCTTAGTAGTAATTTATAGTTGTGTAGTTCACCAAATTCTATTTGATTTGCATAAATATTAAGCATGCAAAACCATATAGTATTATTGTTAATAATATTATTGAGCTGACATTTTGCATATTTTCTCAGCGTGTAGCTACTCATTTCATGCAGTTAATGTTTGCCACTGTTTGTGTCCAGTTCATGGCTATGTAAGTTTATTCAACCTGCTCTCAACTGCGGCCACTTTGGTGCTCTATAATATGTCTATGCGGGTCTGATTTGCATAGTTTGAATTGTTTTGCTTGGCATGGTTTTTGCTGTTTTATTTTTGCATATCTTATCAAATGACGGAATTAGTTCGTTGGCCATGTGTTTTGGGCATTGAGATTTTGTCCATATTCCATATTCCAGTCTGTTTTCTGTCAATCTTATCGGCGACGTGGGAATGCAGCCAATTGCGAATGCATTAAAAGCTTTATGGTGTAACTGAAGACGGCGATCGGTGGTTGACTTGATTGGCAAATGTGCATACTGGGGAAAAGATTTATATGAATATGTATTTAAATTAACTTTGCTTAGGTGCAAAAAACTGGTAGATATAATAATACTCAAAAATGATAATCTATTCCTTTGTAAATATGAGTGAAATAAAACTTGTGTATTTCACCAAATTCCTTTTAATATTAATATAATAATAATAATTTCTTGTAAAATATTCGCTTCTCAATACGAATACTCATAATAGACTTTGTTGATATTTTAAACATCGTGTTAATCATTCGGTAGGGTGTGTACTGTAAAGAAGTGATAAAATCTATGACACACACGGAAATTCTTTTAGTTCATAAGCCAAATATTTGTCACAATTTATATTACATTTATCAGCATTGGCCCATGGCGAATTCAGTGAGCTTGTTTGCGATTTGCTCCTTTCCCAACATTTTTTTTTTGCTGAGACGACAGTGCGTATGCGTAATATGCGTAAATTGCTGTCGCCAGCAATTTGAGCCGTCAAGGAAGCATAAATTAACGCGAATTCCACTTCAGGCATCAGGCCTCCTTCATGCAGAGCCTTTGATCTGTAATTTGATTGATTGAATGAAGCTGGTGAATGGCACTTGAGCGGTGTCGATAAATCAGGGTATCCCTTCATGCTTTTTGACTATGAACGAAAAGCGGAATAGCTGTGTGTATTTTCCCGTTGCTAGAGTAAACAATGATTTTGCCTTTACAAGTTAGCAAAACGATTTTGCATTTCGCACTGCAAATCGTCGAATTGAAGATTTAGACATTTTAGCCCTGTAATGAACAGTTCCGACATTTTAGCTTTTGGCGAAACTTTTACAATGCACCATAAAATGCATTTTAATCGCTTGTTCGCTGAAATCAGGTATAAAGGTGCAATGTGCACAAACAAATAATGAAAACAAAAATGAATAAAGTCATTGCGATGCCTAACGATGCACGGACTGTTAGGCCAATCCCCGGGAATTGTCAATCCCATTTTTGTGTTCTATTCCAAGCGTGTGTTTTATATATATATATATATATTTCCATTGGAAAAAAGCCCTACATTCATATAGTTTCATTTCACACGGAGACAAATGAGTTTCCTGTGTCAACGGCGCCCCAAAAACAATCAAATCGAAACACATTCGTGCCCCACAAATGCAATATTTTATGATACCAATAAAAGGAAACACTTAATCTAAGTCACCATTGACAGTCACATATTTCACTAAGTAGATAAAAATACAGGGATCTGTACTTTAGTTTATATTAGTATAAATATACAAATGTTAAATATATGAACGAATATAAAGTGAATATACACAATATTCAATGGAGAAATTTGAGGAGCATCCTTTATAATATAAACATTCAAAAAAATGAATTAAATTTTTAAATATCCATATAATAGATATTTCCTTTTTATAATTGAGCATTTTGCGAACCAAAATAATTATTCATTGCTATTGGTCGGCGTTCGAATCAAAGCGTTTAAGGACCGAGTCACGTCTTAGATAACCAAAATCGATAGTTGTACAACCATATGGAGCAGCTTTGAGGTATCTTGATTGAAAGTTTCGGTTTATAAAAGTGGCAATTAATTAAAAGCAATTCGTTATTACATTTTATCGATCTTATACCCAACTTATATTCGTAGCTTATTAAAGAACCCAACGAACTGTAGGCAATTTGGGCGCTAATGGCAAGGAAAAGCATTTACTTTCCGATTTATGAGCTTCGAAATGCCATAAAGCTAAGCTATCCGAAATTTACGGAGCTCTCAACTATTTCAGATGCCTGGCGACCAACAATCTGGTGGCTCTACTGGGCATGCTGACCACGACGCTGCTGAAAATGTATCTCTCAAAGGAAGTCCTGCAGTCGTTCATCCGAGTGGATTGCGTGGGACTCGTGGTTTGGCGATTTTTCGGGCTCAGTTCTGGATGCATTGCAGCAGTTATGGCGGCAGAAAGATGGATGGCCCTAGCCAGACCCTTTATCTACCACAAGGTATGAACTTGATTGCAATATATGTATAATAAATAACATTTTCTATGCATTTTATTCTTAGTGTAATTTAATTTTAAGAAATGATACAAGTGCACACAGGAAATCACAAACATTGCCAACTCATTCATTAAATATTCACAAATCTCTCTCGCCATAGCACATTACCTACGAGCTCATTCGCAAGAGCATCAACAGCATTCTGATGATCGCCGTGGTGATCACGTTCCTGCCATTCGTTGGTTTTGGTGCATACATCGATGAATCCAATCCGGACCAGCTTAAGTGTATACGATATCGCGATGCGCCGGGCGTGTGGAACAAGACGTATGCGGTGCTCTTTATGGTCTTCGGTAAGTTCCTATCAAATTGGTTACATTTCAAATATTGGTTTGACCATCGATGTATTCCAGGTACCCTGCTGTGCATTGTAATCGTGGCCTGCAACCTCTTCGTGGCCCACACCTTGCTCTGTGTGATCGGAAGGAGTCGCACGGCCAAGCGGCATATGCACTACGACCTGGTTTCCAGGGACAAAAACAGTGCCATCAGCATCGATCCCGAGAGCAGCAGCGGCACCACCCTCTACCAGACGCAGCTGAGCACCGGAAGCGGAAACAGCCATCGCAGTGTACAGCCGGCAAGGCAGTACAGGCACAGCGTTAGCGTCACAATGGCGGCCACCGACTCCTCGCCAGTCGAGATCAAGTTCGCCAAACTAATGGCGTTCCTCAGCATCTCGTTCGTCATCTGCTGGATGCCACAGATGGTGAGTACTGGGTGGATACCATTCTAAATTTGCAGATAGTAACTACTAAACGTCTAGTATCTTCAACCATTCCGTATTATGCAAATCTAATATAATATAATAAATTTACATGGCCTTTATCAACAATTATCTCCGTTTTAGATCGCCATCCCGTTGGCTATAGCTCCAAATCGGGTGCCCGCATCGAACAAGTTCTTCATCATCGCCGATGTTCTTACGGCGCTGCACTTCACCTCGGATCCGTATGTCTATGTGCTGAGTCGCTCCAAGTCCATCAACTGGTCCTTGCTGGGATGCATTAAGCGCTGGAGGAGCGGATGGCGTCCGGGCGGACTTCGTCGTTCCCAGAGCGACCAGAGCCGCATGCGTACGACGATGACGGAGGCGAATACTCTGGAATTCAACTGATTCCACTCGATCTTCATACCGCGCGCCCCCAAGTACTTAATCCCATTTCCCGACTCAGTGGGATCTCACTCAAAAAAGCGTTTCCGCCTCCCTCGGAGCATTCCTGGGAGTAGCGCCTAGTCTCGTAAGCTGTAAATAATAAATATATCCCACTTCGTATCTTACTATATCAAGGCTTTCCTTATATGTATGTATTCTCACCTCACTTAATCTCTGTACCGATTTGTAGCATTAAATATAACGCTTGTGTAAAAAGATCAAGTATTAAATGTATTAATTGTGCGCTAAAACGAAATCGTACAGCCCTCATAGATATATACACTGCTACTGAATCTCATTTTGCAACAGATTGAAAGCCAATTTCCATATCCCGCAATGACACACAGGAATCGTGTTTGTTTTACAACTAGTTTTGTTTCTTTGTTTATTATAGAATTTATATGATTACATAAGTTGAATGGTTGGTTCGCTGGCTGGCATGGTATGCGTTCTGTGAGGGGGGAATCGGGTAGGTAATACGATTCTCGATTCAATTAACTTGTTTGTGTTCTCCCTCGTCAGTTTCAAGAAGCTTCGTCGTACAAT

>PGR^A1^

gRNA-1: GTAAGTGCAGCTATGCATAG

gRNA-2: GTCGCTCCAAGTCCATCAAC

GCGTGGATGTGGGTGGCCGCTTCGCTTGGGGGTGTCTATGTGCTGTATGCTACATCCGATGCGATCCGACCGGCGGCGGCGCCAGCCGAACTGGCCTCGCTCCGAACTGGCCCCTACCACCACCCCCACGTGTATATGATGGCTTGGATAGCTTGGAGCTATGGGCAGCAGCCGACGAGTGCTCTGCTCGGCACGGTCGGCAGTCATTCTAAATCGACGCCTGGCTGACTGCGGACGCGCTCCTCGGATCGAATCGAATCGCCTTCGAATTGGTCGGTCGTTGGTTGATCAAGTGTCGCGTGCGCTAATCATTAATTAAGTGTCTTAGGAAAAAAGTCCCAATTTGGCTATCGAAACGGGTTTCCATCTACCAGTGCATTTGCGAGCTGCCTTGCCTTTGCGGCAGGCTCATTTATGAAAAAGAAATATCGTTTGCGGCCAGTTAGATTTTCACCTGAATCACCTGCAATCGAACGCAATTATCATACCGGCAAAATGGAAACCACAACACCTGTGCTCGACCTGTTGATGCCGCACAACTCAACTACTGTGGCGCCTCCGAAAGCGCTCTATGCAAATCGAAATCGCCTGATTATTGGTGTTATTATCATGGTTCTGGGCGTTTTTGGCAATTCGCTGGCCCTTTTCATTTTGGCGCGCAAGAAGCTCAACAAAAACAGCAAATACACGCTCATGCTGCGGTAAGTG

Deletion: 3627 bp

TGGTCCTTGCTGGGATGCATTAAGCGCTGGAGGAGCGGATGGCGTCCGGGCGGACTTCGTCGTTCCCAGAGCGACCAGAGCCGCATGCGTACGACGATGACGGAGGCGAATACTCTGGAATTCAACTGATTCCACTCGATCTTCATACCGCGCGCCCCCAAGTACTTAATCCCATTTCCCGACTCAGTGGGATCTCACTCAAAAAAGCGTTTCCGCCTCCCTCGGAGCATTCCTGGGAGTAGCGCCTAGTCTCGTAAGCTGTAAATAATAAATATATCCCACTTCGTATCTTACTATATCAAGGCTTTCCTTATATGTATGTATTCTCACCTCACTTAATCTCTGTACCGATTTGTAGCATTAAATATAACGCTTGTGTAAAAAGATCAAGTATTAAATGTATTAATTGTGCGCTAAAACGAAATCGTACAGCCCTCATAGATATATACACTGCTACTGAATCTCATTTTGCAACAGATTGAAAGCCAATTTCCATATCCCGCAATGACACACAGGAATCGTGTTTGTTTTACAACTAGTTTTGTTTCTTTGTTTATTATAGAATTTATATGATTACATAAGTTGAATGGTTGGTTCGCTGGCTGGCATGGTATGCGTTCTGTGAGGGGGGAATCGGGTAGGTAATACGATTCTCGATTCAATTAACTTGTTTGTGTTCTCCCTCGTCAGTTTCAAGAAGCTTCGTCGTACAAT

>PGR^B1^

gRNA-1: GCCGCACAACTCAACTACTG

gRNA-2: TGCTGGGATGCATTAAGCGC

GCGTGGATGTGGGTGGCCGCTTCGCTTGGGGGTGTCTATGTGCTGTATGCTACATCCGATGCGATCCGACCGGCGGCGGCGCCAGCCGAACTGGCCTCGCTCCGAACTGGCCCCTACCACCACCCCCACGTGTATATGATGGCTTGGATAGCTTGGAGCTATGGGCAGCAGCCGACGAGTGCTCTGCTCGGCACGGTCGGCAGTCATTCTAAATCGACGCCTGGCTGACTGCGGACGCGCTCCTCGGATCGAATCGAATCGCCTTCGAATTGGTCGGTCGTTGGTTGATCAAGTGTCGCGTGCGCTAATCATTAATTAAGTGTCTTAGGAAAAAAGTCCCAATTTGGCTATCGAAACGGGTTTCCATCTACCAGTGCATTTGCGAGCTGCCTTGCCTTTGCGGCAGGCTCATTTATGAAAAAGAAATATCGTTTGCGGCCAGTTAGATTTTCACCTGAATCACCTGCAATCGAACGCAATTATCATACCGGCAAAATGGAAACCACAACACCTGTGCTCGACCTGTTGATGCCGCACAACTCAACTA

Deletion: 3799 bp

TGGGATGCATTAAGCGCTGGAGGAGCGGATGGCGTCCGGGCGGACTTCGTCGTTCCCAGAGCGACCAGAGCCGCATGCGTACGACGATGACGGAGGCGAATACTCTGGAATTCAACTGATTCCACTCGATCTTCATACCGCGCGCCCCCAAGTACTTAATCCCATTTCCCGACTCAGTGGGATCTCACTCAAAAAAGCGTTTCCGCCTCCCTCGGAGCATTCCTGGGAGTAGCGCCTAGTCTCGTAAGCTGTAAATAATAAATATATCCCACTTCGTATCTTACTATATCAAGGCTTTCCTTATATGTATGTATTCTCACCTCACTTAATCTCTGTACCGATTTGTAGCATTAAATATAACGCTTGTGTAAAAAGATCAAGTATTAAATGTATTAATTGTGCGCTAAAACGAAATCGTACAGCCCTCATAGATATATACACTGCTACTGAATCTCATTTTGCAACAGATTGAAAGCCAATTTCCATATCCCGCAATGACACACAGGAATCGTGTTTGTTTTACAACTAGTTTTGTTTCTTTGTTTATTATAGAATTTATATGATTACATAAGTTGAATGGTTGGTTCGCTGGCTGGCATGGTATGCGTTCTGTGAGGGGGGAATCGGGTAGGTAATACGATTCTCGATTCAATTAACTTGTTTGTGTTCTCCCTCGTCAGTTTCAAGAAGCTTCGTCGTACAAT
