# Supplementary material for: Functional characterization of eicosanoid signaling in Drosophila development
Source: PLoS Genet. 2025 May 9;21(5):e1011705. doi: 10.1371/journal.pgen.1011705 (PMC12088517; doi:10.1371/journal.pgen.1011705)
Supplement: S2 Document — (DOCX) [file pgen.1011705.s030.docx]

Intron : xxxx

Exon : xxxx

CDS : xxxx

gRNA : xxxx

>GstS1

AATATCAAATTATCGATAGTACTTCCCCGATAAGATTCTGTTAACACACATTGCTCTACTTTTCTATCAGGGAATTGCATTGAGAAAGCGCAGAGATGGCCGATGAAGCACAAGCACCACCAGCTGAGGGAGCACCACCAGCCGAGGGAGAGGCGCCACCACCCGCCGAGGGAGCCGAGGGTGCCGTCGAGGGCGGAGAGGCCGCTCCACCAGCAGAGCCCGCCGAGCCCATCAAGCACAGCTACACGCTCTTCTACTTCAACGTGAAGGCGTTGGCCGAGCCCCTGCGCTACCTGTTCGCCTACGGCAACCAGGAGTACGAGGATGTGCGCGTCACCCGCGACGAGTGGCCAGCCCTGAAGCCCAGTGAGTGCCATAGTCCATCATTGAGTGGTCAGTGGACTAACCATAATCTACTCTGTCTCATCCAATAGCCATGCCCATGGGACAGATGCCCGTGCTGGAGGTGGACGGCAAGCGTGTGCACCAGAGCATTTCGATGGCTCGATTCCTGGCCAAGACTGTCGGCCTGTGCGGCGCTACTCCGTGGGAGGATCTGCAGATCGACATTGTCGTTGACACCATCAATGACTTCCGTCTAAGTAGCGAACAATGTGTGCCTCAGTTTCATGTTCAGTGTCCTTGATTAACTCTGGAATTTTTAATGTATAGAAATTGCAGTCGTCTCGTACGAGCCGGAGGACGAGATTAAAGAGAAGAAGTTGGTCACCCTGAATGCGGAGGTCATTCCATTCTACCTGGAGAAGCTCGAGCAGACCGTCAAGGACAACGATGGTCACCTGGCTCTGGGCAAGGTGCGTGCTCAAATGAGAACTTGAAACCATACACTCGTTAATTTTGAATGAACAATACTAAGAACCATCGATTCCTTTCACCCACAGCTGACCTGGGCCGACGTCTACTTCGCAGGCATCACCGACTACATGAACTACATGGTCAAGCGCGATCTGTTGGAACCCTACCCAGCTCTGCGCGGAGTCGTGGATGCCGTCAACGCTTTGGAACCGATCAAGGCCTGGATCGAGAAGCGCCCCGTCACCGAGGTCTAAGTTCACCGAACAGGACTTGGGAGGATGGGAAAGCATTGCACAACCACTCAGACACAACCGCACCGCAGAAATGCACACCACCCACATCTGCACACATTTTGTTCCTTTTTGCGGAGGATCCGAGAGGACGGCGGATTTTCGCACGGGGTCATAAACGCGTAGGAGTTTAGCACGTATCACTATGGATAAATCGTATTTGTTCTTAATTTCTTGGTTATAATTCTCTTTGCATGGCGCTATATACATATTTCTATTGGGGTCCGGCAGCGGATCGCCGTTCGTTAGCTTCTTCTGAAATGATGAAAGATAATACTTATTGCACAGTTTATAATATTTTGGCTTACTTTTAGAACATACATTTAGCGCACCTATGCAGACATTTGCTGTACCTATATATATATATATATGCATAAAATACAAAAGCTACGAGTGGTTCATCTTATAGGCGTATTCTATTGCAATTTATTCAATGCGGAGGGCTCAATAAATATTTAAAACAACACCAGCTAAGGCTGAGAAACAATTACAAATTCTTATTTTGTTTTTTATACAACACGCCATCGCGGCCCCAGGAATTTTCTTCGTCTCATCCAGTTCCCTTTTAAGTAGCCATTAAAAAAAGACTCACATCAATAGAACCGGTTTCTCCAAAAAAAAAAAAAACAGACTCTTACTGCCAAATTTCAAATGCACATAATCACATTATTATATACATATTATTTATTCAAATTTGTAATTGTATTTTTTGAATATTTTCACAAAACACTTTTTAATAAACATAACACCTAAAAAGGAATGAGCACTTTTTAAAAGCACGACAACTGGTTTTATTTAATTTTTGTACGATGTATTGGAATAATT

gRNA-1: GCACAAGCACCACCAGCTGA

gRNA-2: AGGTCTAAGTTCACCGAACA

deletion: 983 bp

LoxP: xxxx

3xP3: xxxx

RFP: xxxx

Alpha Tubulin 3’UTR: xxxx

>GstS1^-^

AATATCAAATTATCGATAGTACTTCCCCGATAAGATTCTGTTAACACACATTGCTCTACTTTTCTATCAGGGAATTGCATTGAGAAAGCGCAGAG

AGATCTATAACTTCGTATAATGTATGCTATACGAAGTTATGGTACCGGATCTAATTCAATTAGAGACTAATTCAATTAGAGCTAATTCAATTAGGATCCAAGCTTATCGATTTCGAACCCTCGACCGCCGGAGTATAAATAGAGGCGCTTCGTCTACGGAGCGACAATTCAATTCAAACAAGCAAAGTGAACACGTCGCTAAGCGAAAGCTAAGCAAATAAACAAGCGCAGCTGAACAAGCTAAACAATCGGGCGGCCGCACTAGAGCCGGTCGCCACCATGAGGTCTTCCAAGAATGTTATCAAGGAGTTCATGAGGTTTAAGGTTCGCATGGAAGGAACGGTCAATGGGCACGAGTTTGAAATAGAAGGCGAAGGAGAGGGGAGGCCATACGAAGGCCACAATACCGTAAAGCTTAAGGTAACCAAGGGGGGACCTTTGCCATTTGCTTGGGATATTTTGTCACCACAATTTCAGTATGGAAGCAAGGTATATGTCAAGCACCCTGCCGACATACCAGACTATAAAAAGCTGTCATTTCCTGAAGGATTTAAATGGGAAAGGGTCATGAACTTTGAAGACGGTGGCGTCGTTACTGTAACCCAGGATTCCAGTTTGCAGGATGGCTGTTTCATCTACAAGGTCAAGTTCATTGGCGTGAACTTTCCTTCCGATGGACCTGTTATGCAAAAGAAGACAATGGGCTGGGAAGCCAGCACTGAGCGTTTGTATCCTCGTGATGGCGTGTTGAAAGGAGAGATTCATAAGGCTCTGAAGCTGAAAGACGGTGGTCATTACCTAGTTGAATTCAAAAGTATTTACATGGCAAAGAAGCCTGTGCAGCTACCAGGGTACTACTATGTTGACTCCAAACTGGATATAACAAGCCACAACGAAGACTATACAATCGTTGAGCAGTATGAAAGAACCGAGGGACGCCACCATCTGTTCCTTTAGCGGCCATCGAATTCGAGCTCGCCCACTAAGCGTCGCGCCACTTCAACGCTCGATGGGAGCGTCATTGGTGGGCGGGGTAACCGTCGAAATCAGTGTTTACGCTTCCAATCGCAACAAAAAATTCACTGCAACACTGAAAAGCATACGAAAACGATGAAGATTGTACGAGAAACCATAAAGTATTTTATCCACAAAGACACGTATAGCAGAAAAGCCAAGTTAACTCGGCGATAAGTTGTGTACACAAGAATAAAATCGGCCAGATTCAGTGTTGTCAGAAATAAGAAAACCCCACTATGTTTTTCTTTGCCTTTTCTTTCTCCCAGCGATCATTCATTTCGTGGTGAAAGAACGGGGTCATTGCACGGAGTTTCGACTGCGGGAAAGCAGAGCTGCCGTTCACTTCGTCTATAATTAGCGCTTTCTATTTTCCCCGATTCGGGCCGCTGCTGCGCTTTTCCGCCTGCTGTTTGTGGCAAGTGTAGCAGCAGGCTGTGCACGCAGTGTGGCATGCACTTGGCTTTCCACCGTTGGTATCGATTCTCTGGGACGATGAGTCATTCCTTTCGGGGCCACAGCATAATCGTTGCCAGCTCACCGAAATGGTGACTTCATTTCTTAACTGCCGTCAAGCATGCGATTGTACATACATACATATTTATATATGTACATATTTATGTGACTATGGTAGGTCGATATAATAGCAATCAACGCAAGCAAATGTGTCAGTCCTGCTTACAGGAACGATTCTATTTAGTAATTTTCGTTGTATAAAGTAATTATGTATGTATGTAAGCCCCATAAATCTGAAACAATTAGGCAAAACCATGCGAAGCTCTGCAGATAACTTCGTATAATGTATGCTATACGAAGTTATGCTAGC

AACAGGACTTGGGAGGATGGGAAAGCATTGCACAACCACTCAGACACAACCGCACCGCAGAAATGCACACCACCCACATCTGCACACATTTTGTTCCTTTTTGCGGAGGATCCGAGAGGACGGCGGATTTTCGCACGGGGTCATAAACGCGTAGGAGTTTAGCACGTATCACTATGGATAAATCGTATTTGTTCTTAATTTCTTGGTTATAATTCTCTTTGCATGGCGCTATATACATATTTCTATTGGGGTCCGGCAGCGGATCGCCGTTCGTTAGCTTCTTCTGAAATGATGAAAGATAATACTTATTGCACAGTTTATAATATTTTGGCTTACTTTTAGAACATACATTTAGCGCACCTATGCAGACATTTGCTGTACCTATATATATATATATATGCATAAAATACAAAAGCTACGAGTGGTTCATCTTATAGGCGTATTCTATTGCAATTTATTCAATGCGGAGGGCTCAATAAATATTTAAAACAACACCAGCTAAGGCTGAGAAACAATTACAAATTCTTATTTTGTTTTTTATACAACACGCCATCGCGGCCCCAGGAATTTTCTTCGTCTCATCCAGTTCCCTTTTAAGTAGCCATTAAAAAAAGACTCACATCAATAGAACCGGTTTCTCCAAAAAAAAAAAAAACAGACTCTTACTGCCAAATTTCAAATGCACATAATCACATTATTATATACATATTATTTATTCAAATTTGTAATTGTATTTTTTGAATATTTTCACAAAACACTTTTTAATAAACATAACACCTAAAAAGGAATGAGCACTTTTTAAAAGCACGACAACTGGTTTTATTTAATTTTTGTACGATGTATTGGAATAATT
