# Supplementary material for: Functional characterization of eicosanoid signaling in Drosophila development
Source: PLoS Genet. 2025 May 9;21(5):e1011705. doi: 10.1371/journal.pgen.1011705 (PMC12088517; doi:10.1371/journal.pgen.1011705)
Supplement: S5 Document — (DOCX) [file pgen.1011705.s033.docx]

Intron : xxxx

Exon : xxxx

CDS : xxxx

gRNA : xxxx

>Su(P)

AATAAATATCCCATCCCCAACACATTCTTCAGGGTTACCGTTTAAGGTATGTTCAAAACTTTATGGTATATTCCGGAGCCCTACAAAAGTCTTGGTATTTTTTCACACTGGTCTCATAGTCGCGGCTGATAAGTCGCTAATTTTTGCATAATATTAAGTATAAGTCAAAACAAACGTGCGAATTATGTCATGTATTCGGCTAGCGTCTGCCACCGTTCTGGCAGGGGTCCGTCCCCCGCCGGCAGCAAGCGGAGGTGGAGCCTTGCGCCTGTTGACTAAACGGAATCCGGATGCTGCATTCGCACGCCGCCAGTTTGCTGTAGCGGTTGAAAACGGCGGAGGCAGAAAGAAGCCGAATGGAACTTTCAAGCTCGCAGTTTTGGGGGCCACCGTAGGCGCGGCCACCGGATCGGTGTATACGATGTACCAGCGGTGGACAGACGGCAGCTCGCACAAAGAGCACGAGGAGACGAAGCCGACGCGGCTGGACGGGATTCCGGCTGGAGTGCGGATAACCAAGCGCTATGTCAACCCCAAGGACACGTCCGGTTTGGACATTGTGCTGTTCCAGTTCCAGACGTGTCCCTTCTGTTGCAAGGTGCGCGCTTTCCTCGACTACATGGGCATATCCTATGCGGTGGTCGAAGTGGATGCCGTCCTACGGCAGGACATCCGCTGGTCGTCGGTGAAGAAGGTGCCGATGGTGCTCATCCGACAGCAGGACGGCAAATACGTCCAGATGGTGGACTCAAGTGCCATCATATCCCTTATAGCCACCCACCTGCAGGACAAGCGCACCGACATCGGCGAGCTGGCACAGTTCTATCCGCACACCTCCTTCTTCGACGATGATGGCAAAAAGAAGAACGACATATTGAATAAATACTTCCTCATGTACCGCGAGCATACGCCCAAGGGAGTATCCAAGGAAACGGATGAGTAAGTTAACCAGGGTCAAGTAATCTTTTAATCAAATCTTTTAACCTTGAGTGTCTGTTCTTAGAACCGATCGCAAGTGGAGAAGCTGGGCCGACAGCCACTTGGTGCATCTAATATCGCCCAACTGTTATCAGACCATGGGCGAGTCCCTAGAGACTTTTGAGTGGTTCTCGCAAGCTGGCGAGTGGGACGTTCACTTCCCCAAATGGGAGCGCGACCTGATGGTTTACTGTGGTGCCACCGCCATGTGGGCCATAGCCAAGATGCTGAAGCGCCGTCACGCCCTCAGCGACGACGTCAGGTCGCACATGTACGATGCCCTTGACCAGTGGACCACTGAGCTGAAAAAGAGGAACACGAAATTCATGGGCGGAAAACAGCCAAGTCTGGCTGATCTATCGGTTTTCGGCGTGCTTTCAAGCATGGAGGGGTGTCAGACGTTCAAGGATTGTCTGCAAAACACCAGCATCGGTAAATAAGGTTTCTTTCTCGGGTTTTGTACCTTTTTACGAACTTTTTACATATTTCCGCAGGTAAATGGTTTTATGACGTCAAGGCGTTGGTGGAGAAGAATCGTGGGCAGTTGCAAAGAGAACGTATTGAAAACATGGCCGCTATAGCTTAAAACAAATATTTGCCCTTATTTTATCCAAAGATTGTTGAAAATAACCGAAATGTTCATTAAAATATACATTTTAATCAAATCTAGTTTGCCGTTTCGTTTATGGTATCTGAATGTATATATTTGGTCAAAAACGCGTAAGGAGTGCAAGTTTATGGTACAAATTGGAATTA

gRNA-1: TCACACTGGTCTCATAGTCG

gRNA-2: CGTTGGTGGAGAAGAATCGT

deletion: 1436 bp

LoxP: xxxx

3xP3: xxxx

RFP: xxxx

Alpha Tubulin 3’UTR: xxxx

>Su(P)^-^

AATAAATATCCCATCCCCAACACATTCTTCAGGGTTACCGTTTAAGGTATGTTCAAAACTTTATGGTATATTCCGGAGCCCTACAAAAGTCTTGGTATTTTTTCACACTGGTCTCATAGTCG

AGATCTATAACTTCGTATAATGTATGCTATACGAAGTTATGGTACCGGATCTAATTCAATTAGAGACTAATTCAATTAGAGCTAATTCAATTAGGATCCAAGCTTATCGATTTCGAACCCTCGACCGCCGGAGTATAAATAGAGGCGCTTCGTCTACGGAGCGACAATTCAATTCAAACAAGCAAAGTGAACACGTCGCTAAGCGAAAGCTAAGCAAATAAACAAGCGCAGCTGAACAAGCTAAACAATCGGGCGGCCGCACTAGAGCCGGTCGCCACCATGAGGTCTTCCAAGAATGTTATCAAGGAGTTCATGAGGTTTAAGGTTCGCATGGAAGGAACGGTCAATGGGCACGAGTTTGAAATAGAAGGCGAAGGAGAGGGGAGGCCATACGAAGGCCACAATACCGTAAAGCTTAAGGTAACCAAGGGGGGACCTTTGCCATTTGCTTGGGATATTTTGTCACCACAATTTCAGTATGGAAGCAAGGTATATGTCAAGCACCCTGCCGACATACCAGACTATAAAAAGCTGTCATTTCCTGAAGGATTTAAATGGGAAAGGGTCATGAACTTTGAAGACGGTGGCGTCGTTACTGTAACCCAGGATTCCAGTTTGCAGGATGGCTGTTTCATCTACAAGGTCAAGTTCATTGGCGTGAACTTTCCTTCCGATGGACCTGTTATGCAAAAGAAGACAATGGGCTGGGAAGCCAGCACTGAGCGTTTGTATCCTCGTGATGGCGTGTTGAAAGGAGAGATTCATAAGGCTCTGAAGCTGAAAGACGGTGGTCATTACCTAGTTGAATTCAAAAGTATTTACATGGCAAAGAAGCCTGTGCAGCTACCAGGGTACTACTATGTTGACTCCAAACTGGATATAACAAGCCACAACGAAGACTATACAATCGTTGAGCAGTATGAAAGAACCGAGGGACGCCACCATCTGTTCCTTTAGCGGCCATCGAATTCGAGCTCGCCCACTAAGCGTCGCGCCACTTCAACGCTCGATGGGAGCGTCATTGGTGGGCGGGGTAACCGTCGAAATCAGTGTTTACGCTTCCAATCGCAACAAAAAATTCACTGCAACACTGAAAAGCATACGAAAACGATGAAGATTGTACGAGAAACCATAAAGTATTTTATCCACAAAGACACGTATAGCAGAAAAGCCAAGTTAACTCGGCGATAAGTTGTGTACACAAGAATAAAATCGGCCAGATTCAGTGTTGTCAGAAATAAGAAAACCCCACTATGTTTTTCTTTGCCTTTTCTTTCTCCCAGCGATCATTCATTTCGTGGTGAAAGAACGGGGTCATTGCACGGAGTTTCGACTGCGGGAAAGCAGAGCTGCCGTTCACTTCGTCTATAATTAGCGCTTTCTATTTTCCCCGATTCGGGCCGCTGCTGCGCTTTTCCGCCTGCTGTTTGTGGCAAGTGTAGCAGCAGGCTGTGCACGCAGTGTGGCATGCACTTGGCTTTCCACCGTTGGTATCGATTCTCTGGGACGATGAGTCATTCCTTTCGGGGCCACAGCATAATCGTTGCCAGCTCACCGAAATGGTGACTTCATTTCTTAACTGCCGTCAAGCATGCGATTGTACATACATACATATTTATATATGTACATATTTATGTGACTATGGTAGGTCGATATAATAGCAATCAACGCAAGCAAATGTGTCAGTCCTGCTTACAGGAACGATTCTATTTAGTAATTTTCGTTGTATAAAGTAATTATGTATGTATGTAAGCCCCATAAATCTGAAACAATTAGGCAAAACCATGCGAAGCTCTGCAGATAACTTCGTATAATGTATGCTATACGAAGTTATGCTAGC

ATCCAAAGATTGTTGAAAATAACCGAAATGTTCATTAAAATATACATTTTAATCAAATCTAGTTTGCCGTTTCGTTTATGGTATCTGAATGTATATATTTGGTCAAAAACGCGTAAGGAGTGCAAGTTTATGGTACAAATTGGAATTA
