# Supplementary material for: Functional characterization of eicosanoid signaling in Drosophila development
Source: PLoS Genet. 2025 May 9;21(5):e1011705. doi: 10.1371/journal.pgen.1011705 (PMC12088517; doi:10.1371/journal.pgen.1011705)
Supplement: S6 Document — (DOCX) [file pgen.1011705.s034.docx]

Intron : xxxx

Exon : xxxx

CDS : xxxx

gRNA : xxxx

>cPges

AAATGTTTTGACACAACACTTCAAATTTACGTTACACGTAACAAGCTAACATTTGTGGTTTGAGTACAACTATCGAAGTTTCGCACCCCAGAGCACCGTTCAAATTTAGTCACATCTCCGCAGCGCGCTCGGTAACTCTGTTTCTCATGTCAATTTTGTGCATGACAATTTTGTTCAACAGAAACTGCCTTTTTTTGTAACCAAGTCAGGCTGTCAGTCAGAAGTGTTCCGACTTCGTAAAACATAAACCAACAATTCACAGCAACAACATAATGTCGGCAGCAGCAGGGTGAGTCCACAACCGGCCATCTCAGTGCCAATCGGCGATAGCCGGACCATCTTTTTGTGGCTTCTTTGTGCGTCGCGTGCTTTGTCGTTGCGGCGGCGTCCATTTTCGAACTCCCGGCCGTGAAAAACACATAATTTTCGTTCGGATGACCCTTATTCTCGCGCTCATCCGCCTGGCCAAGCGTTTGTGTCCACGGTGGGCGTGTCCTGTGCGCATCTGCATTGCGATATTTGTGGAACTAATCGGAGGGTCCAGTGTTTGAGGTTATGTTTTCGGCTGGCGGTAATGCCGTGCATTTTCGGCATGGACTTTTGCCTGGGCTTATGGTTTATGCAAATCTAGCTAGCTGGGAAGCTATTTACATAGTTCGTGCACCCGCTTCCAGAAAGTCCGACGCGAGTTGCCATGGCAACGGATCTCGCACAACGACATGAAGCTCTTATTTATGTTTATCCAACTTGATCCTCCATTGCAGCTTGATTCCGCCTCCAGTTTCCTGGGCCCAGCGCAATGACTTGATCTACGTCATCATCGATGTCGAATGCAAGGACATCGAACACAAGTATATGCCCCCATATGTAGTATGTCCATGCTCCTTGCACATCCTAATTCCATATATTTATCCCAAAACAGAGTTACGGAAAAAACCTTCACCTTCAAGGGCGTAAACGTGCTGGATCCGTCGAAGAAGTACGAGGTCACACTGAACTTCCTCCACGAGGTGGATCCCGAGAAGGTGACCAGCAAGAACATTGGCCGCTGCCTGGAATTCACAATACCCAAGAAGGCGGCCGGTCCCTACTGGTCCTCGCTGACCACGGACAAGACCAAGTTGCATTTCCTAAAAGCCAACTTTGCCAAGTGGCGCGATGAGTCCGACGACGAGGAGGGTAAGTTGACATCCTCATCCCCAAGATCTCATCCAGCAAACCCACTCATACAAACGCACTTTGCAGGTGACCAAAAAGACAACAGCATGTTTGGAAATTTCCTTAACAGCCCTGGTGGCGATTGGAACAACAAGTTCGACGATTTCAACGTCGATGACGAGGAGGAGGACTCGGATGACAACATCCCAAGTCTGTCCCAGAACGACGAGGATGACGAGGAGGGCGGCGAGGGTGATAAGGAGAAGAAGCCAGCTGCCTAGACGGTCGCTCAGATGGTGTCTCGCAATTTGGCGTAGTCGTAGCGTTAGGCGTGGCTCTTATACCATAAGTCAGCACACGATCACAACACTCACTCCTCACATACACACCAGAAAAGAAAAAGAAGAAACACTCAATCACTCATAAAACATCCACAAACGCATGCGTACATGAAACACTATTCCAATCGAAGAAAACCACATTTAATATACCTGAATATTATGCGTATTCAGAGGAGAGATGTTAACAAAAAGCAACACAAGGAACAAGATGATTTGGCGCCGACAAGGGAAACCATAAGTTTTACGCTAGAATTTAACAACCAGAGGAAAGCTACAACCAATTTATTGCAAAAATCAGTTTGCAGTTTATTATTTTTGTTTCCTGTAAATAAAGGAAATTAACAATAAAAAAAATGAAAAACTTATATTCGGCTTTTTATTTTCAATATTTTATAAGTTTACAACATTCTATAAATGTTCTAGAAATCTCTAAACTGTTAAATTCGG

gRNA-1: CAGAGTTACCGAGCGCGCTG

gRNA-2: GGAGGGCGGCGAGGGTGATA

deletion: 1326 bp

LoxP: xxxx

3xP3: xxxx

RFP: xxxx

Alpha Tubulin 3’UTR: xxxx

>cPges^-^

AAATGTTTTGACACAACACTTCAAATTTACGTTACACGTAACAAGCTAACATTTGTGGTTTGAGTACAACTATCGAAGTTTCGCACCCCAGAGCACCGTTCAAATTTAGTCACATCTCCGCAG

AGATCTATAACTTCGTATAATGTATGCTATACGAAGTTATGGTACCGGATCTAATTCAATTAGAGACTAATTCAATTAGAGCTAATTCAATTAGGATCCAAGCTTATCGATTTCGAACCCTCGACCGCCGGAGTATAAATAGAGGCGCTTCGTCTACGGAGCGACAATTCAATTCAAACAAGCAAAGTGAACACGTCGCTAAGCGAAAGCTAAGCAAATAAACAAGCGCAGCTGAACAAGCTAAACAATCGGGCGGCCGCACTAGAGCCGGTCGCCACCATGAGGTCTTCCAAGAATGTTATCAAGGAGTTCATGAGGTTTAAGGTTCGCATGGAAGGAACGGTCAATGGGCACGAGTTTGAAATAGAAGGCGAAGGAGAGGGGAGGCCATACGAAGGCCACAATACCGTAAAGCTTAAGGTAACCAAGGGGGGACCTTTGCCATTTGCTTGGGATATTTTGTCACCACAATTTCAGTATGGAAGCAAGGTATATGTCAAGCACCCTGCCGACATACCAGACTATAAAAAGCTGTCATTTCCTGAAGGATTTAAATGGGAAAGGGTCATGAACTTTGAAGACGGTGGCGTCGTTACTGTAACCCAGGATTCCAGTTTGCAGGATGGCTGTTTCATCTACAAGGTCAAGTTCATTGGCGTGAACTTTCCTTCCGATGGACCTGTTATGCAAAAGAAGACAATGGGCTGGGAAGCCAGCACTGAGCGTTTGTATCCTCGTGATGGCGTGTTGAAAGGAGAGATTCATAAGGCTCTGAAGCTGAAAGACGGTGGTCATTACCTAGTTGAATTCAAAAGTATTTACATGGCAAAGAAGCCTGTGCAGCTACCAGGGTACTACTATGTTGACTCCAAACTGGATATAACAAGCCACAACGAAGACTATACAATCGTTGAGCAGTATGAAAGAACCGAGGGACGCCACCATCTGTTCCTTTAGCGGCCATCGAATTCGAGCTCGCCCACTAAGCGTCGCGCCACTTCAACGCTCGATGGGAGCGTCATTGGTGGGCGGGGTAACCGTCGAAATCAGTGTTTACGCTTCCAATCGCAACAAAAAATTCACTGCAACACTGAAAAGCATACGAAAACGATGAAGATTGTACGAGAAACCATAAAGTATTTTATCCACAAAGACACGTATAGCAGAAAAGCCAAGTTAACTCGGCGATAAGTTGTGTACACAAGAATAAAATCGGCCAGATTCAGTGTTGTCAGAAATAAGAAAACCCCACTATGTTTTTCTTTGCCTTTTCTTTCTCCCAGCGATCATTCATTTCGTGGTGAAAGAACGGGGTCATTGCACGGAGTTTCGACTGCGGGAAAGCAGAGCTGCCGTTCACTTCGTCTATAATTAGCGCTTTCTATTTTCCCCGATTCGGGCCGCTGCTGCGCTTTTCCGCCTGCTGTTTGTGGCAAGTGTAGCAGCAGGCTGTGCACGCAGTGTGGCATGCACTTGGCTTTCCACCGTTGGTATCGATTCTCTGGGACGATGAGTCATTCCTTTCGGGGCCACAGCATAATCGTTGCCAGCTCACCGAAATGGTGACTTCATTTCTTAACTGCCGTCAAGCATGCGATTGTACATACATACATATTTATATATGTACATATTTATGTGACTATGGTAGGTCGATATAATAGCAATCAACGCAAGCAAATGTGTCAGTCCTGCTTACAGGAACGATTCTATTTAGTAATTTTCGTTGTATAAAGTAATTATGTATGTATGTAAGCCCCATAAATCTGAAACAATTAGGCAAAACCATGCGAAGCTCTGCAGATAACTTCGTATAATGTATGCTATACGAAGTTATGCTAGC

AGATGGTGTCTCGCAATTTGGCGTAGTCGTAGCGTTAGGCGTGGCTCTTATACCATAAGTCAGCACACGATCACAACACTCACTCCTCACATACACACCAGAAAAGAAAAAGAAGAAACACTCAATCACTCATAAAACATCCACAAACGCATGCGTACATGAAACACTATTCCAATCGAAGAAAACCACATTTAATATACCTGAATATTATGCGTATTCAGAGGAGAGATGTTAACAAAAAGCAACACAAGGAACAAGATGATTTGGCGCCGACAAGGGAAACCATAAGTTTTACGCTAGAATTTAACAACCAGAGGAAAGCTACAACCAATTTATTGCAAAAATCAGTTTGCAGTTTATTATTTTTGTTTCCTGTAAATAAAGGAAATTAACAATAAAAAAAATGAAAAACTTATATTCGGCTTTTTATTTTCAATATTTTATAAGTTTACAACATTCTATAAATGTTCTAGAAATCTCTAAACTGTTAAATTCGG
